# TDP-43 Is Efficiently Transferred Between Neuron-Like Cells in a Manner Enhanced by Preservation of Its N-Terminus but Independent of Extracellular Vesicles

**DOI:** 10.3389/fnins.2020.00540

**Published:** 2020-06-11

**Authors:** Christopher Sackmann, Valerie Sackmann, Martin Hallbeck

**Affiliations:** ^1^Department of Clinical Pathology, Linköping University, Linköping, Sweden; ^2^Department of Biomedical and Clinical Sciences, Linköping University, Linköping, Sweden

**Keywords:** TDP-43, extracellular vesicles, C-terminus, N-terminus, cell-to-cell, protein transfer, amyotrophic lateral sclerosis, frontotemporal lobar degeneration

## Abstract

The misfolding of transactive response DNA-binding protein (TDP-43) is a major contributor to the pathogenesis of TDP-43 proteinopathies, including amyotrophic lateral sclerosis and frontotemporal lobar degeneration with TDP-43 inclusions, but also plays a role in other neurodegenerative diseases including Alzheimer disease. It is thought that different truncations at the N- and C-termini of TDP-43 contribute to its misfolding and aggregation in the brain, and that these aberrant TDP-43 fragments contribute to disease. Despite this, little is known about whether different truncation events influence the protein’s transmissibility between cells and how this cell-to-cell transfer occurs. In this study, we use a well-established cellular model to study the efficiency by which full-length and truncated TDP-43 fragments are transferred between neuron-like cells. We demonstrate that preservation of the N-terminus of TDP-43 enhances its transmissibility between cells and that this protein transmission occurs in a manner exclusive of extracellular vesicles, instead requiring cellular proximity for efficient propagation. These data indicate that the N-terminus of TDP-43 might be a useful target in the generation of therapeutics to limit the spread of TDP-43 pathology.

## Introduction

Transactive response DNA-binding protein 43 (TDP-43) is known to play a key role in the pathogenesis of canonical TDP-43 proteinopathies such as amyotrophic lateral sclerosis (ALS) and frontal temporal lobar dementia (FTLD) but is also frequently observed in more common neurodegenerative diseases (NDs) including tauopathies, synucleinopathies, and up to 57% of Alzheimer’s disease (AD) patients (Josephs et al., 2014a; Mishima et al., 2017; Koga et al., 2018; Robinson et al., 2018). TDP-43 is highly conserved phylogenetically, and knockout of the TDP-43 encoding gene, TARDBP, results in embryonic lethality early in embryogenesis (Kraemer et al., 2010; Sephton et al., 2010; Wu et al., 2010). In its native state, TDP-43 performs critical transcription modulating functions relating to the stability and splicing of RNA, including the inclusion/exclusion of introns and exons thought to be important in both normal function as well as in the pathogenesis of neurodegeneration (Ling et al., 2015; Gu et al., 2017; Sun et al., 2017).

A number of disease-related posttranslational modifications have been described, including hyperphosphorylation at the C-terminus and truncation events resulting in the aggregation prone, cytotoxic fragments found in the inclusions of ALS and FTLD brains (Berning and Walker, 2019). C-terminal fragments of TDP-43 are often considered to be the main truncation event in pathogenesis; however, there is growing evidence that truncation events targeting other parts may be important to the disease process as well. These include the N-terminus (Shiina et al., 2010; Chang et al., 2012; Zhang et al., 2013; Afroz et al., 2017), which is necessary for TDP-43 self-assembly (and therefore its native RNA splicing functions), but also distinct regions within the protein core, primarily the RNA recognition motifs (RRM) and glycine-rich domain (GRD), which influence its ability to form aggregates (Yang et al., 2010; Shimonaka et al., 2016).

TDP-43 proteinopathies share a number of similarities with other NDs, including progressive involvement of interconnected brain regions as disease progression occurs, as well as the ability of TDP-43 aggregates to seed further aggregation (Nonaka et al., 2013; Porta et al., 2018). The direct, neuron-to-neuron transmission of disease-related proteins, including amyloid-β (Aβ), α-synuclein, and tau have been observed (Clavaguera et al., 2009; Luk et al., 2012; Nath et al., 2012; Domert et al., 2016; Sackmann et al., 2017, 2019). However, comparatively few studies have investigated TDP-43, particularly with focus on which cellular mechanisms might facilitate its transfer between cells (Feiler et al., 2015; Smethurst et al., 2016; Zeineddine et al., 2017; Porta et al., 2018; Laferrière et al., 2019). Perhaps most importantly, there is little known about the transmissibility of truncated forms of TDP-43, which are thought to facilitate cytotoxicity, aggregation, and therefore pathogenesis, in TDP-43 proteinopathies (Zhang et al., 2009).

To elucidate whether TDP-43 and TDP-43 truncated fragments are transferred directly between cells and, if so, which regions of the protein are important to the transfer process, we generated a number of cell lines expressing different fragments of TDP-43. Using our well-established, highly differentiated three-dimensional coculture cellular model, we studied the ability of each expression construct in their propensity to transfer between cells and their downstream effects upon the recipient cell.

## Materials and Methods

### Generation of TDP-43 Expressing Cell Lines

**FIGURE 1:**
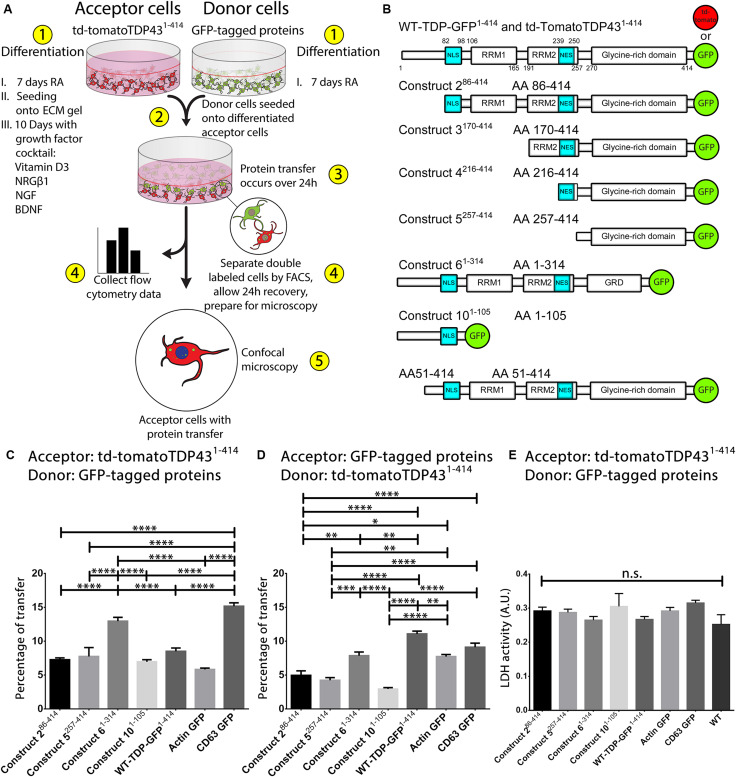
Preservation of the N-terminus of TDP-43 promotes its transmissibility between cells. A visualization of the coculture model is provided in **(A)**. Cells are differentiated in ECM gel with growth factors according to the scheme described in **(A)** prior to formation of the coculture. After 24 h of coculture, cells are analyzed and separated using FACS and prepared for microscopy. Experiments involve the use of full-length TDP-43 and TDP-43 truncated fragments, all of which are fused to fluorescent proteins at the C-terminus **(B)**. Two full-length TDP-43 proteins were used, td-tomatoTDP43^1– 414^ and WT-TDP43^1– 414^ (GFP), whereas all TDP-43 protein fragments were fused with EGFP. All fragments of TDP-43 transferred directly from donor cells (GFP) to td-tomatoTDP43^1– 414^ acceptor cells with differing degrees of efficiency **(C)**. Full-length td-tomatoTDP43^1– 414^ donor cells transferred protein at similar levels to TDP-43 fragment expressing acceptor cells **(D)**. Construct 6^1– 314^ was transferred most readily among the different TDP-43 fragments, indicating that preservation of the N-terminus (and/or loss of the extreme C-terminus) of TDP-43 is influential to the transmissibility of TDP-43 between cells. Cell toxicity, as measured by LDH release, indicated that the cocultures did not exhibit cytotoxicity over 24 h **(E)**. Images corresponding to data set C can be found in Figure 2, whereas images from data set D are presented in Supplementary Figure S3. Full ANOVA tables are available in Supplementary Tables S3, S4. Data are presented as the proportion of all detected acceptor cells that are double labeled, provided as the mean ± SEM. Significance determined by one-way ANOVA with Tukey *post hoc* test, *n* = 6–10 **(C)**, and *n* = 4–7 **(D)**. *p < 0.05, **p < 0.01, ***p < 0.001, ****p < 0.0001, n.s., not significant. BDNF, brain-derived neurotrophic factor; NRGβ1, neuregulin-β1; NGF, nerve growth factor; RA, retinoic acid; FACS, fluorescence-activated cell sorting; NLS, nuclear localization sequence; NES, nuclear export sequence; RRM, RNA recognition motif.

Plasmids expressing full-length TDP-43 and fragments of TDP-43 were obtained from Addgene. Plasmids deposited to Addgene by Yang et al. (2010). A list of all the plasmids used in this study is provided in Supplementary Table S1. SH-SY5Y cells were transfected using Lipofectamine LTX (Invitrogen, Gothenburg, Sweden) according to manufacturer’s instructions. G418 (600 μg/mL) was used over several generations to generate stable expression in each cell line. The TDP-43 AA51–414 plasmid was generated from full-length TDP-43 using the Q5 Site-Directed Mutagenesis Kit (NEB, Solna, Sweden) with the primers: 5′-ATGAGAGGTGTCCGGCTGGTAGAAG-3′ and 5′-GGCGATCGCGGCGGCAGA-3′ and confirmed by sequencing. Three of the constructs (construct 3^170– 414^, construct 4^216– 414^, and construct AA51–414) were highly cytotoxic and resulted in cell death within 72 h, so we were unable to generate stable expressing cell lines with these constructs. The naming scheme of the constructs was adapted from Yang et al. (2010). All TDP-43 constructs used in the study are fused at the C-terminus with their respective fluorophores as outlined in Figure 1B. An actin–green fluorescent protein (GFP) cell line was generated for use as a negative control in order to measure baseline protein transfer. Likewise, a CD63–GFP (a marker of multivesicular bodies) cell line was developed for use as a positive control as CD63-positive vesicles have been previously shown to efficiently transfer intercellularly using this cellular model (Sardar Sinha et al., 2018). Expression of each construct by respective cell lines were confirmed by Western blot (Supplementary Figure S1).

### Cell Culture, Differentiation, and Coculture

Unless stated otherwise, all cell culturing was performed using MEM with GlutaMAX (Gibco, Gothenburg, Sweden), supplemented with 10% fetal bovine serum (Gibco, Gothenburg, Sweden), 50 μg/mL streptomycin (Lonza, Gothenburg, Sweden), 50 U/mL penicillin (Lonza, Gothenburg, Sweden), and 2 mM L-glutamine (Lonza, Gothenburg, Sweden). Differentiation and coculture were performed as previously described (Agholme et al., 2010). In order to differentiate the acceptor cell population, SH-SY5Y cells (ECACC; Sigma-Aldrich, Stockholm, Sweden) are predifferentiated in 10 μM retinoic acid (RA; Sigma-Aldrich) for 7 days. These cells are then resuspended onto extracellular matrix (ECM) gel (Corning, Gothenburg, Sweden) at a 1:2 ratio [ECM:serum-free media (SFM)]. The cells in three-dimensional gel suspension are then further differentiated for 10 days using the following growth factor cocktail in SFM: brain-derived neurotrophic factor (BDNF, 50 ng/mL; PeproTech, Stockholm, Sweden), neuregulin β1 (10 ng/mL; R&D Systems, Abingdon, United Kingdom), nerve growth factor (10 ng/mL; R&D Systems), and vitamin D3 (24 nM; Sigma-Aldrich). In parallel, donor SH-SY5Y cells are generated by differentiation with 10 μM RA for 7 days. The donor cells are resuspended onto the fully differentiated, ECM suspended acceptor cells, and this coculture was incubated for 24 h before analysis. To ensure consistency between replications, donor cells were seeded at a ratio of 7:10 (donor:acceptor). In order to detect protein transfer between cells, donor and acceptor cells expressing different fluorescent proteins were used. For example, donor cells expressing GFP were used when acceptor cells expressed td-tomato and vice versa. Data corresponding to these experiments are depicted in their respective figures as the proportion of acceptor cells that were double labeled, indicative of protein transmission. A flowchart of the differentiation and experimental procedures can be found in Figure 1A.

To definitively demonstrate that passage of TDP-43 and its fragments were transferred in both directions during coculture (donor cell to acceptor cell, but also vice versa), additional experiments were performed involving the use of Qdot-800 (Qtracker 800 Cell Labeling Kit; Invitrogen) to further label the donor cell population. Qtracker was chosen because it has minimal impact on cellular processes and does not leak out of cells and because of its favorable emission spectrum. The coculture process was performed similarly to that described above with minor modifications. In this circumstance, donor cells are differentiated in the same way but, on day 6 of differentiation, were supplemented with Qtracker-800 according to manufacturer’s instructions. The following day, cells were sorted to collect those that had taken up Qdot-800 nanocrystals and resuspended onto the fully differentiated (including growth factor cocktail), ECM-suspended acceptor cells, where they were left for 24 h before performing further analysis. Data corresponding to these experiments are depicted in their respective figures as the proportion of protein transfer events that have occurred through “anterograde transfer” (donor cell to acceptor cell) and “retrograde transfer” (acceptor cell to donor cell). A flowchart of the differentiation and experimental processes for the Qdot-labeled coculture studies can be found in Figure 3A.

For Transwell coculture experiments, donor and acceptor cells were cultured in the same manner as described previously. Rather than seeding donor cells directly onto the acceptor cells in ECM gel, they were plated into Transwell plate inserts with a 0.4-μm pore (Falcon, Gothenburg, Sweden), using the same 7:10 (donor:acceptor) cell ratio used in all experiments. This configuration permits donor and acceptor cells to secrete cell products into a shared extracellular milieu, while maintaining a physical barrier between both populations. Data corresponding to these experiments are depicted in their respective figures as the proportion of acceptor cells that were double labeled, indicative of protein transmission. A flowchart of the experimental process for the Transwell experiments can be found in Figure 4F.

Treatment of acceptor cells with extracellular vesicles (EVs) was done as previously described (Sardar Sinha et al., 2018). In order to mitigate any possibility that EV motility could be inhibited by the 3D ECM gel, acceptor cells in this scenario were differentiated for 7 days in RA on conventional laboratory plastics. RA differentiated SH-SY5Y cells readily take up other aggregated proteins (Domert et al., 2014, 2016; Sackmann et al., 2017). Cells were plated onto 24-well plates at 25,000/cm^2^ and incubated with 1,000 ng EV isolated protein (see separate EV isolation section) for 24 h. Following this incubation, cells were analyzed by flow cytometry. Data corresponding to these experiments are depicted in their respective figures as the proportion of wild-type (WT) unlabeled acceptor cells that were fluorescently labeled, indicative of protein transmission. A flowchart of the experimental process for the EV experiments can be found in Figure 4E.

When measuring cytotoxicity in cocultures, lactate dehydrogenase (LDH) assay kit (Pierce, Gothenburg, Sweden) was used according to manufacturer’s instructions, measured at 490 and 650 nm.

### EV Isolation

TDP-43 expressing cell lines were differentiated for 7 days with RA. After the differentiation process, cells were washed thoroughly with phosphate-buffered saline (PBS) to ensure the removal of potentially contaminating EVs of fetal bovine serum origin. Following this important washing step, the media was replaced with fresh SFM and incubated for 48 h to allow release of EVs into the media. This conditioned media was collected for isolation by differential ultracentrifugation as described previously (Sardar Sinha et al., 2018). Culture supernatants were initially spun at 2,000 × *g* for 10 min to remove cellular debris. The resulting supernatant was sequentially centrifuged: 10,000 × *g* (30 min), 100,000 × *g* (120 min); the pellet resuspended/washed in PBS and spun again at 100,000 × *g* (120 min; all steps performed at 4°C). The final pellet was resuspended in PBS. To ensure that equal amounts of EV isolated protein were added to acceptor cells in downstream experiments, the protein content of the EV resuspension was quantified using Quant-iT Protein Assay Kit (Invitrogen) according to manufacturer’s instructions. Cells were plated onto 24-well plates at 25,000/cm^2^ and incubated with 1,000 ng of isolated EV protein for 24 h.

To confirm the validity of our EV preparations from cell culture, we isolated and analyzed EVs from human tissues. Fresh-frozen postmortem brain samples of temporal neocortex from healthy (non-demented) donors were provided by the brain bank at Uppsala University, Sweden. The collection and use of the tissue were approved by the Regional Ethical Committee in Uppsala, Sweden (2005/103, 2005-06-29; 2009/089; 2009-04-22). Isolation of brain exosomes from freshly frozen human brain tissues (250 mg) was performed by tissue dissociation with papain (20 U/mL, 15 min at 37°C; Sigma-Aldrich) followed by filtration through 40-μm mesh filter (BD Biosciences, Stockholm, Sweden) and 0.2-μm syringe filter (Thermo, Gothenburg, Sweden). The crude exosomes were then isolated by differential centrifugation as described above.

### Western Blotting

Confirmation of EV preparations and TDP-43 expression in each construct-expressing cell line was performed by Western blotting. For EV preparations, EVs were pelleted and lysed in RIPA buffer [50 mM Tris-HCl, 150 mM NaCl, 1% Triton-X 100, 0.5% sodium deoxycholate, and 0.1% sodium dodecyl sulfate (SDS), supplemented with phosphatase inhibitor (PhosStop; Roche, Solna, Sweden) and protease inhibitor (Halt; Thermo)], followed by vigorous vortexing. Extracellular vesicle lysates were mixed with Laemmli loading buffer and dithiothreitol (DTT), boiled at 95°C for 5 min, and loaded into a 4–20% SDS ClearPage gel (CBS Scientific, Gothenburg, Sweden), and transferred onto nitrocellulose membranes (iBlot; Invitrogen). Extracellular vesicle blots were visualized using SuperSignal West Femto ECL substrate (Thermo). For confirmation of TDP-43 expression in construct-expressing cell lines, the preparation was the same as above, except for the use of a different lysis buffer (62.5 mM Tris-HCl, 10% glycerol, and 2% SDS in MilliQ H_2_O) and ECL reagent (Clarity ECL Substrate; Bio-Rad, Solna, Sweden). A list of antibodies used in this study indicating manufacturer and dilutions is provided in Supplementary Table S2.

### Flow Cytometry, Cell Sorting, and Microscopy

To liberate cocultured cells embedded in ECM gel, cells were treated with Corning Recovery Solution (Corning) according to manufacturer’s instructions. Analysis and cell sorting were carried out simultaneously using a FACSAria III (BD Biosciences), equipped with the following laser and filter configurations for the detection of each fluorophore: GFP: 488 nm laser, 530/30 emission filter; td-tomato: 561 nm laser, 610/20 emission filter; Qdot-800: 405 nm laser, 780/60 emission filter. Gating strategies are provided in Supplementary Figure S6. Where possible, all acceptor cells that had protein transfer were collected, replated, and incubated overnight to allow reattachment to glass cover slides. The following day, cells were fixed with 4% paraformaldehyde (PFA) and mounted with ProLong Gold with DAPI (Invitrogen). Images were acquired with a Zeiss, Stockholm, Sweden LSM700 confocal microscope equipped with a Zeiss Axio Observer inverted microscope using a 63× objective with a 1.4 numerical aperture oil immersion lens. A 405-nm laser was used for the excitation of DAPI, GFP, and Qdot-800, whereas a 555-nm laser was used for the excitation of td-tomato. Images of each individual fluorophore were acquired on independent tracks (allowing the use of lasers and emission filters optimized for each individual fluorophore) to minimize spectral overlap of fluorophores. Image processing was done with Zen Lite (Zeiss). The Coloc 2 plugin for ImageJ (NIH, Bethesda, MD, United States) was used to determine colocalization of construct 6^1– 314^ and CD63–GFP with td-tomatoTDP43^1– 414^. Images presented are representative of each respective experimental permutation, in which approximately 80,000 events were analyzed per *n*.

## Results

### Full-Length TDP-43 and Its Truncated Fragments Efficiently Transfer Between Cells

While previous studies have demonstrated the propagation of TDP-43 pathology in the brain, it remains unclear by which manner this propagation occurs, as reviewed in Hanspal et al. (2017). In order to determine whether full-length TDP-43 and truncated TDP-43 are transferred between cells, we adapted our well-established coculture model (Figure 1A), which upon differentiation gives rise to highly differentiated cells that display several mature neuronal phenotypes (Agholme et al., 2010; Domert et al., 2014, 2016; Sackmann et al., 2017, 2019).

This coculture model has been used previously to demonstrate the cell-to-cell transfer of Aβ and α-synuclein but had not been used to examine the transfer of TDP-43 between cells. In order to assess the suitability of this coculture model for the study of cell-to-cell transmission of TDP-43, we initially confirmed that full-length TDP-43 was efficiently transferred from donor cells to acceptor cells (Figure 1C). After using the full-length TDP-43 constructs to confirm that TDP-43 was indeed transferred between cells in this model, we next aimed to study whether truncated (N- and C-terminal) TDP-43 proteins could be transferred, because of their importance in the pathogenesis of TDP-43 proteinopathies. We developed a number of cell lines that stably expressed full-length TDP-43 or truncated TDP-43 constructs (Figure 1B) for use in our coculture model. The cells generated for this study display similar phenotypes (including subcellular localization) as reported in previous studies using cells generated with these constructs (Yang et al., 2010). Images of undifferentiated cell lines can be found in Supplementary Figure S2. Donor cells that stably express a TDP-43 construct were seeded onto acceptor cells that stably express full-length TDP-43 tagged at the C-terminus with td-tomato (td-tomatoTDP43^1– 414^). After 24 h, the coculture was analyzed by flow cytometry (Figure 1C). We observed that all TDP-43 fragments transferred between cells efficiently; however, construct 6^1– 314^ and WT-TDP-GFP^1– 414^ (full-length TDP-43 fused to GFP) were found to be transferred most readily compared to the other TDP-43 constructs. We utilized CD63–GFP (a marker of EVs) and actin–GFP as positive and negative control cell lines, respectively. Both of these control cell lines also demonstrated cell to cell transfer, with CD63–GFP showing the greatest degree of transfer among all permutations. The full analysis of variance (ANOVA) table corresponding to the transfer experiment in Figure 1C is provided in Supplementary Table S3.

In addition to these initial transfer experiments, we also examined this phenomenon with the donor and acceptor cell pairs reversed. That is, the TDP-43 fragment (GFP)–expressing cells were differentiated into the acceptor cells, whereas the td-tomatoTDP43^1– 414^ cells were used as donor cells. In this way, we validate that the transfer is a bona fide event while also illuminating whether this transfer is skewed in one direction or another (do TDP-43 fragments transfer to full-length TDP-43, full-length TDP-43 transfer to the TDP-43 fragments, or both?). In this reversed scenario, we noted a similar trend for TDP-43 transfer (Figure 1D). While transfer was slightly diminished in general compared to Figure 1C, we observed that the construct 6^1– 314^ and WT-TDP-GFP^1– 414^ acceptor cells displayed the highest amount of protein transfer relative to the other TDP-43 fragments. The full ANOVA table corresponding to the transfer experiment in Figure 1D is provided in Supplementary Table S4. To ensure that transfer values were not influenced by the release of cellular contents due to cell death, we measured cytotoxicity by LDH, which showed no statistical significance between each of the different constructs (Figure 1E). Together, these data indicate that both construct 6^1– 314^ and full-length TDP-43 enhance the cell’s propensity to transfer proteins. Furthermore, it implies that there is a substantial amount of protein transfer in both directions (from donor cell to acceptor cell, but also acceptor cell to donor cell). We speculate that the minute differences in transfer between Figures 1C,D are related to differences in “anterograde” (donor cell to acceptor cell) and “retrograde” (acceptor cell to donor cell) transfer. However, using this experimental design, the readout for transfer in both directions involves the collection and observation of double-labeled (GFP + td-tomato) cells. Therefore, we cannot assert with certainty which direction the protein transfer occurs using this experimental paradigm. To circumvent this, we developed a model that further labels the donor cells, evaluated later in this study.

**FIGURE 2:**
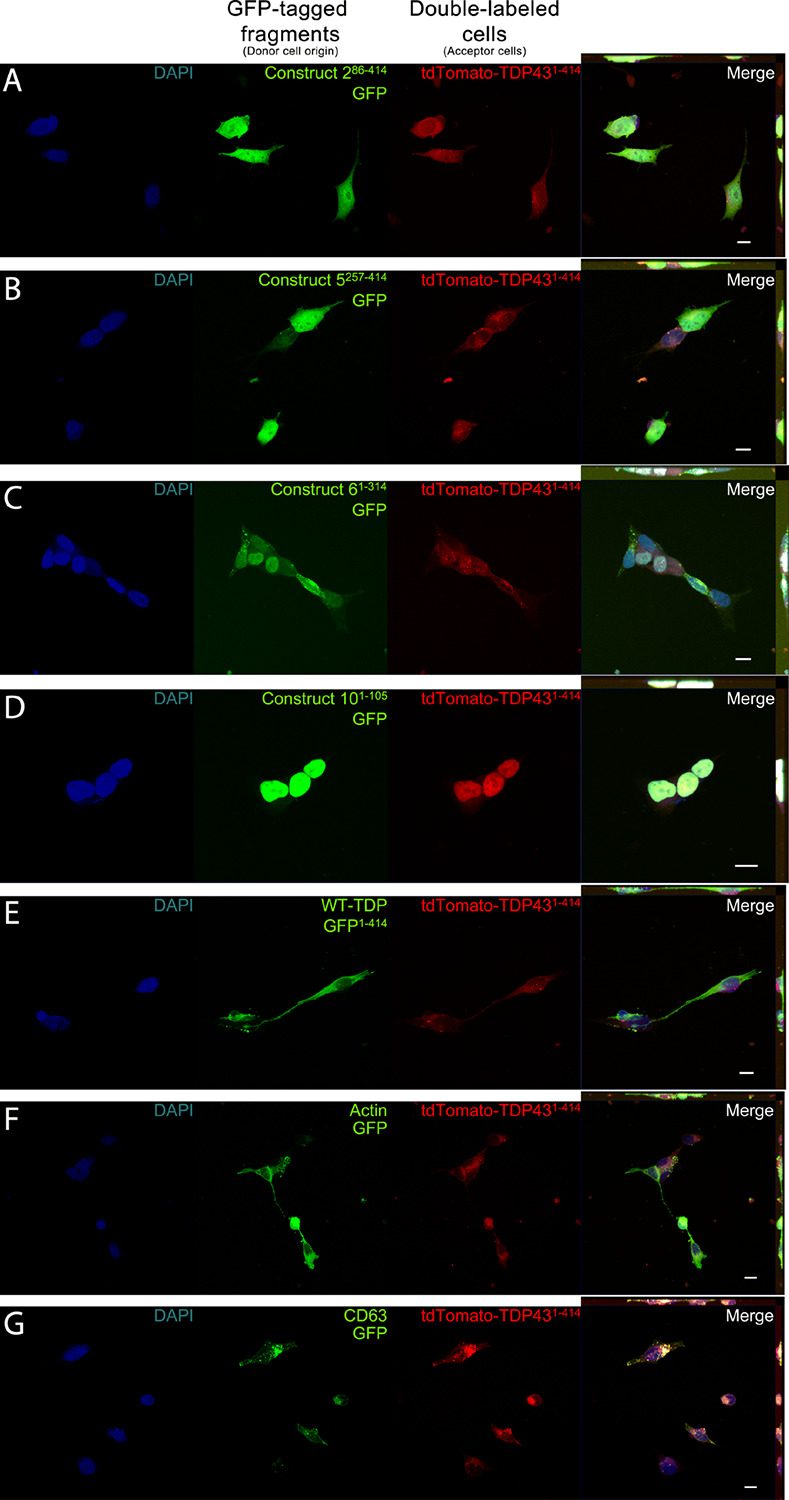
Representative images depicting cellular localization of GFP-tagged TDP-43 fragments (donor cells; green) after transfer to full-length td-tomatoTDP43^1– 414^ acceptor cells (acceptor cells; red). Acceptor cells (td-tomatoTDP43^1– 414^), which also displayed GFP positivity, were sorted and fixed for confocal microscopy. Association of full-length TDP-43 from acceptor cells was observed with varying degrees to each TDP fragment **(A–E)** as well as with actin **(F)** and CD63 **(G)**. Construct 10^1– 105^ **(D)** shows localization exclusively to the nucleus, whereas constructs 2^86– 414^, 5^257– 414^, 6^1– 314^, and WT-TDP-GFP^1– 414^ (**A–C,E**, respectively) could be found in the cytoplasm, as well as the nucleus. The final column contains Z-projections. Scale bar = 10 μm.

In addition to confirming that protein transfer occurs between donor and acceptor cells, we also determined the compartmentalization of the transferred proteins, as aberrant localization of TDP-43 is a marker of TDP-43 pathology. As an RNA-binding molecule, TDP-43 is natively found in the nucleus, whereas pathogenic TDP-43 can be found in the cytoplasm (Neumann et al., 2006). Each TDP-43 fragment was predominantly found in the same subcellular compartments presynaptically and postsynaptically. Construct 2^86– 414^ was found primarily in the nucleus, but also to a lesser extent uniformly distributed throughout the cell soma (Figure 2A). Similarly, construct 5^257– 414^ localized prominently to the nucleus, but was also found distributed to a lesser degree within the cell body (Figure 2B). While construct 2^86– 414^ has the nuclear localization sequence (NLS) and nuclear export sequence (NES) intact, construct 5^257– 414^ has both regions omitted, perhaps having a deleterious effect upon its localization, whereas construct 6^1– 314^ displays two unique phenotypes: cytoplasmic and nuclear puncta (Figure 2C). While many punctate structures could be found in the cytoplasm (indicative of TDP-43 pathology), they did not reliably colocalize with the full-length td-tomatoTDP43^1– 414^ puncta of the acceptor cells (Pearson correlation coefficient = 0.48). Construct 10^1– 105^ was localized entirely to the nucleus, which was anticipated given that it contains only a short N-terminus fragment containing the NLS and no NES (Figure 2D). Of particular interest was the full-length TDP-43–expressing construct WT-TDP-GFP^1– 414^ (Figure 2E), which displayed almost entirely cytoplasmic localization. This signifies that the transfer process of full-length TDP-43 in itself might elicit some pathogenic process during the transfer operation. Lastly, our control cell lines, actin–GFP and CD63–GFP, appeared as expected, with actin localizing throughout the cell (Figure 2F) and CD63 puncta seen throughout the cytoplasm (Figure 2G). CD63–GFP displayed a high degree of colocalization with td-tomatoTDP43^1– 414^ (Pearson correlation coefficient = 0.82). This enhanced colocalization of CD63 with td-tomatoTDP43^1– 414^ suggests that, at the very least, TDP-43 is sequestered to subcellular compartments containing burgeoning EVs. The observed colocalization between TDP-43 and CD63 suggests that transfer of TDP-43 between cells might occur via an EV-mediated mechanism, which is investigated later in this study. The images provided in Figure 2 correspond to the transfer data provided in Figure 1C. Images corresponding to Figure 1D (td-tomatoTDP43^1– 414^ as the donor cells, GFP-tagged fragments as the acceptor cells) are provided in Supplementary Figure S3.

We also performed these transfer experiments using the C-terminal TDP-43 fragment constructs 3^170– 414^, 4^216– 414^, and AA51–414. All three of these proteins were highly cytotoxic, with transfected cells dying within 72 h, eliminating the possibility of generating stably expressing cells lines. Transiently transfected cells with constructs 3^170– 414^, 4^216– 414^, and AA51–414 all demonstrated the ability to transfer to acceptor cells, but these events were comparatively very rare because of the low transfection efficiency and lethality of these constructs, so were not included in the data.

### TDP-43 Transfers Bidirectionally With Similar Efficiency—TDP-43 Fragment to Full-Length TDP-43 and Vice Versa

**FIGURE 3:**
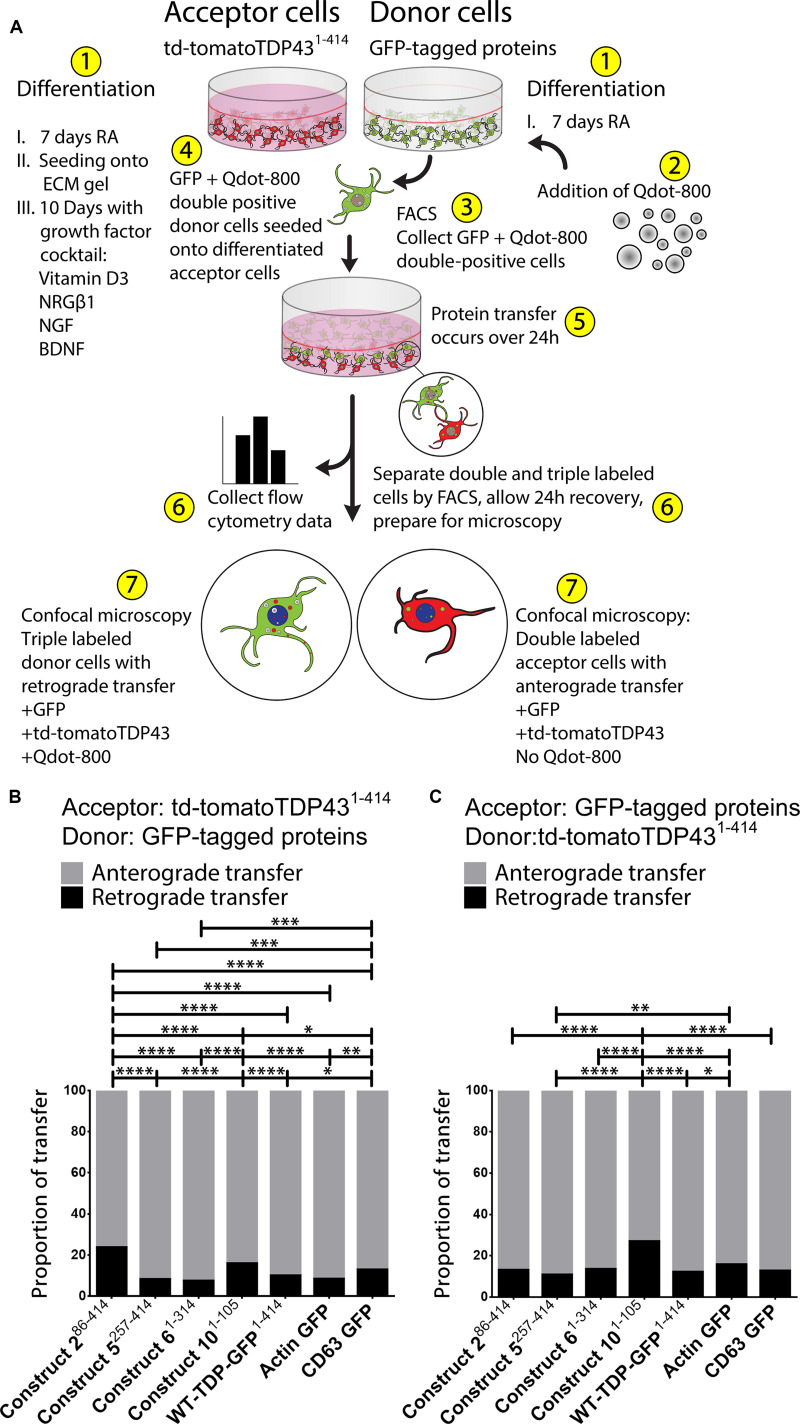
The majority of TDP-43 transfers occur from donor to acceptor cells (“anterograde”); however, there is a lesser degree of transfer from the acceptor to the donor cells (“retrograde”). A visualization of the Qdot-800 coculture model is provided in **(A)**. Cells are differentiated in ECM gel with growth factors according to the scheme described. Prior to formation of the coculture, donor cells (shown here as GFP) were further labeled with Qdot-800 and selected by FACS to collect double-positive donor cells (+GFP and +Qdot-800) and plated directly onto fully differentiated acceptor cells. After 24 h of coculture, donor cells displaying retrograde transfer and acceptor cells displaying anterograde transfer were analyzed and separated using FACS and prepared for microscopy. Of the total transfer events, the majority of TDP-43 was transferred from donor to acceptor cells (anterograde; gray) (**B,C**, presented as proportions of detected transfer events). TDP-43 and TDP-43 truncated fragments also transferred from acceptor cells to donor cells (retrograde; black); however, retrograde transfer was comparatively minimal. Cell images corresponding to these data can be found in Supplementary Figure S4 (for data set B), and Supplementary Figure S5 (for data set C). Full ANOVA tables are provided in Supplementary Tables S5, S6. Data are presented as mean with significance determined by one-way ANOVA with Tukey *post hoc* test, *n* = 3–4 for each permutation. *p < 0.05, **p < 0.01, ***p < 0.001, ****p < 0.0001, ns, not significant. The data represent the detected retrograde and anterograde transfer events as a proportion of all detected transfer events. BDNF, brain-derived neurotrophic factor; NRGβ1, neuregulin-β1; NGF, nerve growth factor; RA, retinoic acid; FACS, fluorescence-activated cell sorting.

Although the above experiments support the notion that full-length TDP-43 and truncated TDP-43 fragments have the ability to transfer between cells (albeit with differing degrees of efficiency), we wanted to further investigate the direction of transfer in order to unequivocally confirm that transmission of TDP-43 between cells is not restricted only to full-length protein or fragmented protein. To investigate if the transfer events occurred primarily “anterograde” (donor cell to acceptor cell), primarily “retrograde” (acceptor cell to donor cell), or a combination of both, we modified the coculture model by further labeling the donor cells with Qtracker-800 prior to the formation of the coculture. These Qdot-800 nanocrystals do not interfere with cellular processes and are not passed between adjacent cells (Desplats et al., 2009), making them suitable for distinguishing our donor and acceptor cell populations. By introducing this additional labeling of the donor cells, we were able to reliably track the direction of protein transfer in the coculture. After donor cell differentiation, we used fluorescence-activated cell sorting (FACS) to collect the double-labeled donor cells (+GFP and +Qdot-800) and seeded these cells directly onto the differentiated acceptor cells, at the same ratio (donor cells:acceptor cells) used previously. After the 24 h incubation of coculture, we analyzed and collected both populations for microscopy: triple-labeled donor cells with retrograde transfer (+GFP, +Qdot-800, and +td-tomato) and double-labeled acceptor cells with anterograde transfer (+GFP, +td-tomato, and No Qdot-800). In this way, we were able to track both the transmission of TDP-43 between cells, but also determine in which direction the transfer occurred. A flowchart of this experiment is provided in Figure 3A.

Using this Qdot-800 coculture system, we determined that, as a proportion of all TDP-43 transfer events, the majority of TDP-43 is transferred from donor to acceptor cell (“anterograde”), regardless of which TDP-43 fragment was examined (Figure 3B). As in Figure 1C, constructs 2^86– 414^ and 10^1– 105^ showed the least amount of anterograde transfer, compared to constructs 6^1– 314^ and WT-TDP-GFP^1– 414^, which showed the greatest degree of anterograde transfer among the TDP-43 constructs. Like our previous experiments, we also reversed the donor/acceptor pairs (Figure 3C). Much like in the previous transfer experiment (Figure 1D), construct 10^1– 105^ expressing acceptor cells displayed the least amount of anterograde transfer (full-length td-tomatoTDP43^1– 414^ proteins transferred toward construct 10^1– 105^ acceptor cells) in this experimental paradigm, therefore skewing the proportion of transfer events toward retrograde (acceptor cell to donor cell) in this circumstance. Taking Figures 1C,D together with Figures 3B,C, these data indicate that protein transfer of full-length TDP-43 and TDP-43 truncated fragments occurs in both directions, demonstrating that TDP-43 transmission between cells is not restricted only to full-length protein or fragmented protein. Subcellular localization of TDP-43 and TDP-43 truncated fragments (presynaptically and postsynaptically) was analagous to those presented in Figure 2 and Supplementary Figure S3. Cell images corresponding to these experiments, Figures 3B,C, are provided in Supplementary Figures S4, S5, respectively. Full ANOVA tables corresponding to the transfer experiments in Figures 3B,C are provided in Supplementary Tables S5, S6, respectively.

### Transfer of TDP-43 and TDP-43 Fragments Is Independent of Extracellular Vesicles but Requires Physical Proximity Between Cells

We have previously used this cellular coculture model to show that Aβ and α-synuclein can be transferred between cells via EVs (Sardar Sinha et al., 2018; Sackmann et al., 2019). Given the colocalization observed between of TDP-43 with CD63–GFP (Figure 2G and Supplementary Figures S3G, S4G, S5G), along with its prominent transfer propensity (Figures 1C,D), we investigated whether EVs might be a mechanism mediating the transfer of TDP-43 between cells.

**FIGURE 4:**
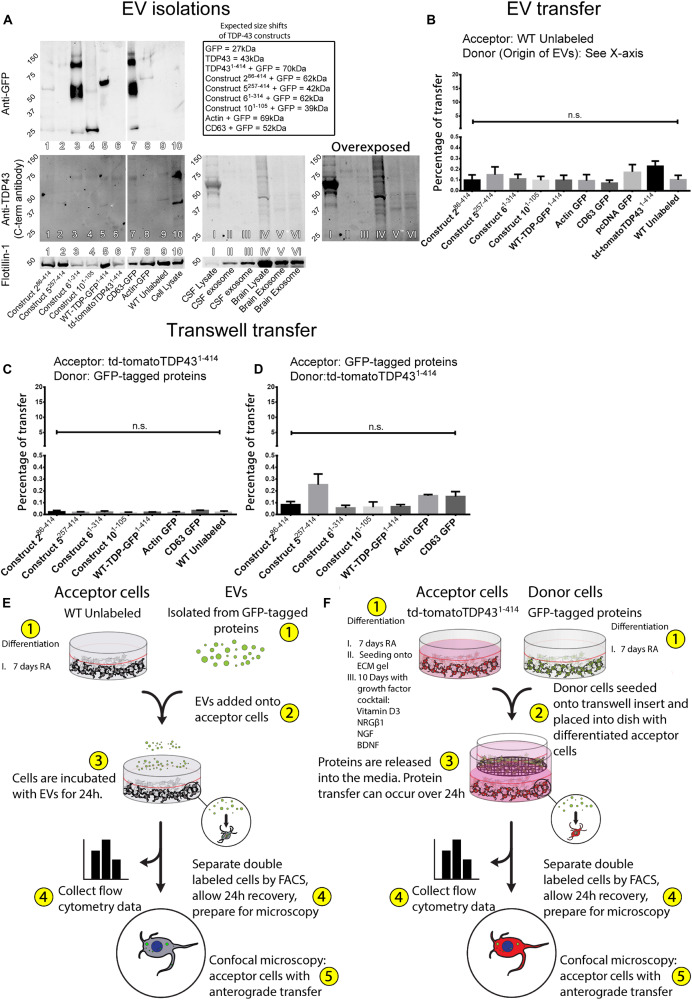
Transfer of TDP-43 and TDP-43 fragments requires physical connectivity between cells. Extracellular vesicles isolated from stably expressing TDP-43 cell lines and human tissues indicate that the TDP-43 fragments are incorporated into EVs, albeit with a low degree of efficiency **(A)**. The TDP-43 antibody used here recognizes an epitope at the extreme C-terminus of the WT-TDP-GFP^1– 414^, which is absent in some constructs or obfuscated by the C-terminal GFP tags but can be observed with the anti-GFP antibody (e.g., construct 6^1– 314^). Isolated EVs were added directly onto WT SH-SY5Y cells but failed to transfer any appreciable protein **(B)**. Further, donor and acceptor cells were physically separated during coculture using Transwell inserts, allowing the distribution of cell products within a shared medium while creating a physical barrier between the cells **(C,D)**, but proteins also fail to transfer by this method. Graphical representations of the experimental setup are shown in **(E,F)**. Full ANOVA tables of **(B–D)** are available in Supplementary Tables S7–S9. Data in **(B)** are presented as the proportion of all detected cells that displayed detectable fluorescence, provided as mean ± SEM. Data in **(C,D)** are presented as the proportion of all detected acceptor cells that are double labeled, provided here mean ± SEM. Significance determined by one-way ANOVA with Tukey *post hoc* test, *n* = 6–9 **(B)**, and *n* = 3 **(C,D)**. *p < 0.05, **p < 0.0, ***p < 0.001, ****p < 0.0001, ns, not significant.

Extracellular vesicles were isolated from the conditioned media of each TDP-43 construct–expressing cell line using the differential ultracentrifugation method. The presence of EVs in the isolations was first confirmed by the detection of flotillin-1 by Western blot, and the preparations were then analyzed for TDP-43 (Figure 4A). Because of the conjugation of GFP to the C-terminus of each construct, commonly used antibodies such as clone TDP2H4, which target a C-terminal epitope, fail to detect the TDP-43-GFP conjugated proteins (Figure 4A). Using this C-terminal specific antibody to measure TDP-43 in EVs resulted in the detection of faint bands of TDP-43 at 43 kDa (singlet) and ≈80 kDa (doublet). The singlets and doublets detected in this blot originate from the endogenously produced TDP-43 exclusive of the introduced TDP-43–GFP–tagged proteins, determined by the comparable expression patterns observed between the cell lysate, WT unlabeled, actin–GFP, and CD63–GFP lanes compared to the construct-expressing cell lines. Nevertheless, the TDP-43 detected by the TDP2H4 antibody identifies the endogenously produced TDP-43 in each cell line at similar molecular weights and expression levels compared to those observed in EVs isolated from human cerebrospinal fluid (CSF) and brain (Figure 4A, see human tissue EV isolations). The similarities between the human tissue EV isolations and the cell-derived EVs validate both the EV isolation method and the notion that TDP-43 is incorporated into EVs (albeit at very minute levels).

In order to measure whether TDP-43 and TDP-43 fragments from each respective construct-expressing cell line were incorporated into EVs, we probed this same blot for GFP, which is conjugated to the C-terminus of each construct (excluding td-tomatoTDP43^1– 414^, which expresses a C-terminal td-tomato tag) (Figure 4A). GFP was detected in EVs isolated from the conditioned media of construct 2^86– 414^, 6^1– 314^, and the full-length WT-TDP-GFP^1– 414^, each appropriately weight shifted for its respective TDP-43 fragment + GFP tag (see inset). As expected, td-tomatoTDP43^1– 414^ was not detected here as it does not express GFP. Construct 10^1– 105^ shows very little if any staining at the appropriate size (≈39 kDa). The absence of construct 10^1– 105^ in the EV-enriched fraction could be related to its hyperlocalization to the nucleus (Figure 2D), likely relating to its expression of the TDP-43 NLS but not NES, reducing its exportation from the nucleus to the cytoplasm and contributing to its relatively low transfer rates (Figures 1C,D). Likewise, construct 5^257– 414^ was not detected in EVs at the expected molecular weight (≈42 kDa). Construct 5^257– 414^ lacks the TDP-43 NLS and the NES and displayed similar levels of protein transfer compared to construct 10^1– 105^ (Figures 1C,D, construct 5^257– 414^ vs. construct 10^1– 105^ displayed no statistical significance). Conversely, construct 6^1– 314^ was detected abundantly in EVs, with a prominent smear appearing at the predicted weight, ≈60 kDa, as well as a fainter band at ≈120 kDa and a fainter band still greater than 150 kDa. We postulate that these bands correlate to construct 6^1– 314^ singlets, doublets, and triplets, respectively. The full-length TDP-43–expressing cell line WT-TDP-GFP^1– 414^ also demonstrated a significant propensity for incorporation into EVs, showing a prominent band at the expected molecular weight of ≈70 kDa. Interestingly, the two most readily TDP-43 proteins incorporated into EVs (construct 6^1– 314^ and WT-TDP-GFP^1– 414^) were also the two constructs that demonstrated the highest amount of protein transfer (Figures 1C,D). CD63–GFP displayed prominent bands at the expected size (≈52 kDa), and its relative abundance further validates the isolation method. Finally, actin–GFP also demonstrated incorporation into EVs with a detectable band at ≈69 kDa. The detection of actin–GFP in EVs was expected as actin is a known component of EVs. Taken together, an intact N-terminus (and/or cleaved C-terminus) is shown to be important to TDP-43’s proclivity to be transmitted between cells (Figure 1), but also promotes their incorporation into EVs (Figure 4A).

The isolated EVs were then added to WT RA differentiated cells 24 h before analysis by flow cytometry. In this circumstance, acceptor cells were differentiated with RA without ECM gel, to eliminate the possibility that EV motility would be hindered by the extracellular gel matrix. Remarkably, despite the detection of TDP-43 within EVs generated by these cells, analysis of the acceptor cells indicated that no appreciable protein transfer had occurred for any of the constructs (Figure 4B). A visualization of the experimental design and execution of the EV experiments is provided in Figure 4E.

Having observed that EVs do not play a significant role in TDP-43 transfer, we investigated the importance of other mediators of protein transfer that work at a distance. We performed transfer experiments involving the physical separation of donor and acceptor cells using Transwell cell culture inserts. This method precludes direct physical connections between the donor and acceptor cells while allowing unrestricted passage of extracellular mediators throughout a shared medium. Here, donor and acceptor cells were differentiated in the same manner as in previous experiments; however, rather than seeding the donor cells onto the gel acceptor cells in the transfer experiments, donor cells were instead plated onto a Transwell membrane. Downstream analysis was performed in the same manner as with previous experiments. A visualization of the experimental design and execution is provided in Figure 4F. Surprisingly, this method of physical separation also eliminated any protein transfer between the cells (Figure 4C), which is in corroboration with insufficient protein transfer by direct EV treatment. As in previous transfer experiments, this was further confirmed when the donor/acceptor pairs were reversed, such that the full-length td-tomatoTDP43^1– 414^ was used as a donor cell and the TDP-43 fragments assumed the role as acceptor (Figure 4D). Full ANOVA tables corresponding to the transfer experiments in Figures 4B–D are provided in Supplementary Tables S7–S9, respectively.

These data show that, although TDP-43 and fragments of TDP-43 are found in EVs, they are not a prominent contributor to the total transfer of TDP-43. Our data also indicate that there is a degree of preferential sorting of TDP-43 fragments into EVs, such that C-terminal fragments are not efficiently packaged into EVs, whereas TDP-43’s containing an intact N-terminus (and/or absence of the extreme C-terminus) are packaged into EVs more favorably. This hypothesis, that the N-terminus of TDP-43 is important for its packaging into EVs, coincides with the observations that the N-terminus was related to increased protein transfer, alluding to the importance of the TDP-43 N-terminus in this system. Further, these data indicate that transfer of TDP-43 and TDP-43 truncated fragments occurs by a mechanism dependent upon cellular proximity.

## Discussion

The accumulation and deposition of misfolded TDP-43 throughout interconnected brain regions have been well described in FTLD, ALS, AD, and other NDs (Brettschneider et al., 2013; Josephs et al., 2014b; James et al., 2016), leading to speculation that TDP-43 might spread directly between cells (Nonaka et al., 2013; Porta et al., 2018). TDP-43 has been demonstrated to recruit further protein misfolding of naive TDP-43 in a prion-like manner both *in vitro* (Smethurst et al., 2016) and *in vivo* (Porta et al., 2018), a common pathological feature of NDs. While previous studies have reported the cell-to-cell transmission of TDP-43 (Nonaka et al., 2013; Feiler et al., 2015; Zeineddine et al., 2017; Porta et al., 2018; Laferrière et al., 2019) and aggregation potential (Yang et al., 2010; Zhang et al., 2013; Shimonaka et al., 2016), these studies have not reported the transmissibility of truncated TDP-43, a critical component in the pathogenesis in TDP-43 proteinopathies. Further, there remains uncertainty as to which region(s) of TDP-43 are involved in the generation of aggregates and therefore pathogenesis (Yang et al., 2010; Nonaka et al., 2013; Zhang et al., 2013; Feiler et al., 2015; Shimonaka et al., 2016; Zeineddine et al., 2017; Zacco et al., 2018).

In this study, we have adapted our well-established 3D coculture model (Agholme et al., 2010; Domert et al., 2014, 2016; Sackmann et al., 2017, 2019) to show that TDP-43 and its truncated fragments are transferred directly between cells and that this protein transfer is not facilitated by components released by cells, which can be found in conditioned media such as EVs or other mediators. This cellular model has been used extensively to characterize cell-to-cell transmission of different ND-related proteins including α-synuclein and Aβ, as well as the uptake of exosomes containing these proteins (Nath et al., 2012; Domert et al., 2016; Sackmann et al., 2017, 2019; Sardar Sinha et al., 2018). The differentiation process used in these experiments results in a number of neuron-like phenotypes including the expression of mature tau isoforms and neuronal markers (SV2 and NeuN) not otherwise observed in SH-SY5Y cells (Agholme et al., 2010), making this model more physiologically relevant than previous studies using HEK-293 or undifferentiated SH-SY5Y cells. By using stably expressing cell lines rather than adding exogenous protein, we eliminate any bias toward uptake and recycling mechanisms, which may be otherwise overrepresented in the protein transfer.

We show that construct 6^1– 314^ was transferred most readily between cells and showed some degree of colocalization to the full-length TDP-43 protein in acceptor cells. This construct lacks the C-terminus of TDP-43, indicating that the C-terminus is either inconsequential to protein transfer or is inhibitory. Indeed, constructs that express the C-terminus, but lack the N-terminus (constructs 2^86– 414^ and 5^257– 414^), display significantly reduced propensity for protein transfer. Previous studies allege that the RRM2 region of TDP-43, particularly AA216–257, was required for TDP-43 aggregation (Yang et al., 2010), whereas others demonstrate the importance of AA274–353 GRD to the aggregation process (Shimonaka et al., 2016). Construct 5^257– 414^, which lacks the RRM2 sequence, shows no increased proclivity for transfer relative to construct 2^86– 414^, which retains the RRM2 sequence. Similarly, the AA274–353 GRD is intact in both constructs 2^86– 414^ and 5^257– 414^, but again no appreciable difference in transfer potential was observed with these constructs, nor was there any perceivable inclination toward aggregation with these two constructs, presumably from the lack of a misfolded TDP-43 seed to initiate aggregation templating. Interestingly, construct 6^1– 314^, which harbors both the RRM2 and the GRD up to AA314, showed the highest amount of protein transfer, but also readily formed punctate structures. In a study by Shimonaka et al. (2016), they suggest that two separate sections of the GRD were sufficient for protein aggregation, AA274–313 and AA314–353. Because construct 6^1– 314^ harbors the AA274–313 region of the GRD, spontaneously forms puncta, and is most readily transferred, we speculate that this region might also play some role in TDP-43’s ability to transfer between cells. While the RRM2 region may be of importance toward TDP-43 aggregation, it does not appear to be particularly influential to its transfer. It should be noted that we attempted to generate stable cell lines, which included these regions of interest (construct AA51–414, construct 3^170– 414^, and construct 4^216– 414^), but these constructs proved to be highly lethal, where transfection with each of these plasmids led to cell death within 72 h. While we were unable to create stable cell lines with these constructs, we were able to perform transfer studies by transient transfection and confirmed that they also successfully transferred their proteins to acceptor cells, but given the rarity of the construct AA51–414, construct 3^170– 414^ and construct 4^216– 414^ fragment–expressing cells surviving the transfection, coculture, and FACS process, we did not include these data.

Because the C-terminal fragments all resulted in significantly diminished TDP-43 protein transfer, it would be expected that construct 10^1– 105^ would transfer most efficiently; however, we observed that it is comparable to the rate by which constructs 2^86– 414^ and 5^257– 414^ were transferred. It is likely that, because this protein fragment is so tightly localized to the nucleus by the presence of the NLS while also lacking the NES, this impedes its ability to transfer with greater efficiency. This is further exemplified by the equitable levels of transfer between the aforementioned construct 10^1– 105^ and construct 5^257– 414^, which lacks both the NLS and NES, and is found primarily in the nucleus. In a previous report, the extreme N-terminus (AA1–10) was found to be most crucial for the native function of TDP-43, which requires homodimerization, as well as a necessity in the formation of TDP-43 aggregates (Zhang et al., 2013). We observed that cell lines that lacked this AA1–10 region (constructs 2^86– 414^ and 5^257– 414^) had diminished protein transfer propensity, but also that these lines did not form punctate structures. In contrast, construct 6^1– 314^ but also the full-length TDP-43 cell lines displayed the highest degree of protein transfer between cells, as well as the spontaneous formation of puncta in donor and acceptor cells in all scenarios. Taken together, we suggest that these data signify the importance of the N-terminus, presumably AA1–50, as the region most involved in promoting TDP-43 transfer, whereas preservation of the C-terminus (AA315–414) inhibits TDP-43 transfer.

Recently, evidence has emerged indicating that EVs may be involved in the spreading of misfolded proteins involved in ND pathogenesis (Rajendran et al., 2006; Danzer et al., 2012; Asai et al., 2015; Sardar Sinha et al., 2018; Sackmann et al., 2019). TDP-43 has been found in the EV fraction of transiently transfected HEK-293 cells (Feiler et al., 2015), as well as EVs isolated from CSF and brains of patients with TDP-43 proteinopathies (Feneberg et al., 2014; Ding et al., 2015; Iguchi et al., 2016). In our own studies using this same 3D coculture cell model, we have shown that Aβ can be transferred between cells via EVs (Sardar Sinha et al., 2018). In the present study, the inability of TDP-43 to be transferred between cells was particularly unexpected given that these experiments were carried out using fivefold EV concentrations relative to those used previously (Sardar Sinha et al., 2018). In the current study, however, the preparations were not fortified with a pan-EV membrane dye. As EVs derived from SH-SY5Y express low levels of CD63 relative to other EV-associated proteins (Gustafsson et al., 2018), it is conceivable that the amount of CD63–GFP EVs in these preparations is not sufficient for the detection by FACS without labeling the entire membrane. Even so, we have demonstrated the presence of a small amount of TDP-43 in EVs from CSF and brains of healthy subjects, as well as in EVs released from several of the TDP-43–expressing SH-SY5Y cell lines. In agreement with our observation that transfer of TDP-43 fragments was enhanced by the preservation of the N-terminus of TDP-43 (and/or loss of C-terminus), we show that these constructs (construct 6^1– 314^ and the full-length WT-TDP-GFP^1– 414^) also displayed a higher degree of incorporation into EVs. However, ultimately EVs were not shown to be an efficient mechanism of transfer of fragmented or full-length TDP-43. In concordance with these data, when donor cells were physically separated from acceptor cells but shared a common extracellular environment, TDP-43 transfer was similarly abolished, in accordance with previous reports (Pokrishevsky et al., 2016). Taken together, this strongly indicates that while TDP-43 and its fragments are present in EVs, EVs are not a significant route of intercellular TDP-43 protein transfer. Nevertheless, it remains a possibility that transmission of TDP-43 via EVs could occur with concentrations much greater than those necessary for the propagation of other ND-related proteins, which we have used as a benchmark in this study. The data presented here support the previous suggestion that physical connectivity between cells is required for transfer of TDP-43, possibly across the synaptic cleft itself (Feiler et al., 2015).

## Conclusion

This study shows that fragmented TDP-43 and full-length TDP-43 are transferred efficiently between neuron-like cells. While full-length TDP-43 and all N- and C-terminally truncated fragments displayed some degree of transfer between cells, those with intact N-termini were transferred significantly more readily. This cell-to-cell protein transfer is mediated by a mechanism dependent on physical proximity; thus, while full-length TDP-43 and truncated fragments were found within EVs, they were unable to transfer the TDP-43 proteins efficiently to recipient cells. These findings provide novel insights into the disease propagation mechanism of TDP-43 proteinopathies, which can be important in the search for disease-modifying therapeutics for TDP-43 proteinopathies.

## Data Availability Statement

All datasets generated for this study are included in the article/Supplementary Material.

## Ethics Statement

Fresh-frozen post-mortem brain samples of temporal neocortex from healthy (non-demented) donors were provided by the brain bank at Uppsala University, Sweden. The collection and use of the tissue was approved by the Regional Ethical Committee in Uppsala, Sweden (2005/103, 2005-06-29; 2009/089; 2009-04-22). The patients/participants provided their written informed consent to participate in this study.

## Author Contributions

CS and MH: study concept and design, interpretation of data, and manuscript preparation. CS and VS: experimental work. CS: analysis of data. All authors critically read and approved the manuscript.

## Conflict of Interest

The authors declare that the research was conducted in the absence of any commercial or financial relationships that could be construed as a potential conflict of interest.

## Supplementary Material

FIGURE S1 Western blots demonstrating the expression of each TDP-43 fragment in stably transfected cell lines. Each TDP-43 fragment is expressed in cells and demonstrate bands at expected weight shifts for fusion proteins. TDP-43 has many different possible cleavage events, which are shown in the full blots provided here. Further, while many TDP-43 cleavage events occur, cleavage of GFP from the TDP-43-GFP proteins are minimal. The TDP-43 antibody used for here binds to an epitope near the N-terminus of the protein. As such, constructs missing this region have a diminished appearance (e.g., construct 5^257– 414^). There is also the possibility for detection of the endogenous TDP-43, as well as possible fragment events in both the overexpressed and endogenous TDP-43 (e.g., td-TomatoTDP43^1– 414^). See Figure 1B for a visualization of each construct used in this study.

FIGURE S2 Representative images depicting cellular localization of constructs from each transfected cell line without protein transfer, displaying phenotypes similar to those previously reported. Similar to the localization observed in co culture experiments, Construct 2^86– 414^ localized predominantly to the nucleus, Construct 5^257– 414^ is found primarily within the nucleus but also distributed throughout the cell body. Construct 6^1– 314^ displays cytoplasmic and nuclear puncta, Construct 10^1– 105^ is exclusively expressed in the nucleus, and the full-length constructs WT-TDP-GFP^1– 414^ and td-tomatoTDP43^1– 414^ are found distributed throughout the cell body and nucleus. Actin is localized throughout the cell body and CD63 displays puncta throughout the cell. Scale bar = 50 μm.

FIGURE S3 Representative images depicting cellular localization of full-length td-tomatoTDP43^1– 414^ (donor cells; red) after transfer to cells expressing GFP-tagged TDP-43 fragments (acceptor cells; green). Acceptor cells which were double positive (+GFP and +td-tomatoTDP43^1– 414^) were sorted and fixed for confocal microscopy. Like Figure 2, association of full-length TDP-43^1– 414^ from donor cells was observed with varying degrees to each TDP fragment **(A–E)** as well as with actin **(F)** and CD63 **(G)**. Construct 10^1– 105^ **(D)** shows localization exclusively to the nucleus, whereas constructs 2^86– 414^, 5^257– 414^, 6^1– 314^, and WT-TDP-GFP^1– 414^ (**A,B,C,E**, respectively) could be found in the cytoplasm as well as the nucleus. The final column contains *Z*-projections. Scale bar = 10 μm.

FIGURE S4 Representative images depicting subcellular localization of TDP-43 in donor cells exhibiting retrograde transfer. GFP-tagged protein expressing donor cells [+GFP (green), +Qdot-800 (white), +td-tomatoTDP43^1– 414^ (red)] display triple labeling after retrograde transfer of td-tomatoTDP43^1– 414^ originating from acceptor cells. Acceptor cells (td-tomatoTDP43^1– 414^) transferred proteins to the donor cells (+GFP and +Qdot-800; retrograde transfer), albeit less efficiently than donor to acceptor cells (anterograde; see Figure 3B). Cells which were triple-positive (+GFP, +Qdot-800, and +td-tomatoTDP43^1– 414^) were selected using FACS and fixed for confocal microscopy. This data indicates that full-length TDP-43 and truncated TDP-43 both harbor the ability to transfer between cells. Subcellular localization of TDP-43 fragments **(A–D)**, full-length TDP-43 **(E)**, actin **(F)** and CD63 **(G)** are akin to those observed in previous experiments (see Figure 2 and Supplementary Figures S3, S5). The final column contains *Z*-projections. Scale bar = 10 μm.

FIGURE S5 Representative images depicting subcellular localization of TDP-43 fragments in donor exhibiting retrograde transfer. td-tomatoTDP43^1– 414^ expressing donor cells [+td-tomatoTDP43^1– 414^ (red), +Qdot-800 (white), and +GFP (green)] display triple labeling after retrograde transfer of GFP-tagged TDP-43 truncated proteins originating from acceptor cells. Acceptor cells (GFP) transferred proteins to the donor cells (+td-tomatoTDP43^1– 414^ and +Qdot-800; retrograde transfer), albeit less efficiently than donor to acceptor (see Figures 3B,C). Cells which were triple-positive (+GFP, +td-tomatoTDP43^1– 414^, and +Qdot800) were selected using FACS and fixed for confocal microscopy. This data indicates that most of the protein transfer occurs from donor to acceptor cell (anterograde), however, there is a modest amount of protein transfer from the acceptor cells to the donor cells (retrograde). Subcellular localization of TDP-43 fragments **(A–D)**, full-length TDP-43 **(E)**, actin **(F)** and CD63 **(G)** are akin to those observed in previous experiments. The final column contains *Z*-projections. Scale bar = 10 μm.

FIGURE S6 Gating strategies for cell sorting by FACS and representative population graphs. Unlabeled cells were first used to determine the live cell population and td-tomato (PE-Texas Red) and GFP (Alexa Fluor 488) negative cells using cut-off values determined by the authors. This was followed by gating strategies using only td-tomato or GFP-tagged acceptor cells. **(A)** Gating strategies for Figures 1C,D and **(B)** for Figures 4B–D. **(C)** Figures 3B,C utilized Qdot labeled donor cells that were first gated and then followed the gating strategies as the previous sets. Acceptor cells with protein transfer were then used to determine the percentage of transfer and sorted for confocal microscopy.

TABLE S1 List of plasmids used in the study.

TABLE S2 List of antibodies used in the study.

TABLE S3 ANOVA table corresponding to Figure 1C. The mean values provided here are the percentage of td-tomatoTDP43^1– 414^ acceptor cells that are double labeled (acceptor cells with positive transfer).

TABLE S4 ANOVA table corresponding to Figure 1D. The mean values provided here are the percentage of GFP-tagged protein expressing acceptor cells that are double labeled (acceptor cells with positive transfer).

TABLE S5 ANOVA table corresponding to Figure 3B. The mean values provided here are: of the total transfer events measured, those that were anterograde (td-tomatoTDP43^1– 414^ acceptor cells that are double labeled). The retrograde values are the difference, out of 100, and therefore give the same, but inverse, tabular results.

TABLE S6 ANOVA table corresponding to Figure 3C. The mean values provided here are: of the total transfer events measured, those that were anterograde (GFP-tagged protein acceptor cells that are double labeled). The retrograde values are the difference, out of 100, and therefore give the same, but inverse, tabular results.

TABLE S7 ANOVA table corresponding to Figure 4B. The mean values provided here are the percentage of WT SH-SY5Y acceptor cells that are fluorescently labeled.

TABLE S8 ANOVA table corresponding to Figure 4C. The mean values provided here are the percentage of td-tomatoTDP43^1– 414^ acceptor cells that are double labeled (acceptor cells with positive transfer).

TABLE S9 ANOVA table corresponding to Figure 4D. The mean values provided here are the percentage of GFP-tagged protein expressing acceptor cells that are double labeled (acceptor cells with positive transfer).

## References

[B1] Afroz T. Hock E. M. Ernst P. Foglieni C. Jambeau M. Gilhespy L. A. B. (2017). Functional and dynamic polymerization of the ALS-linked protein TDP-43 antagonizes its pathologic aggregation. *Nat. Commun.* 8 1–14. 10.1038/s41467-017-00062-0 28663553 PMC5491494

[B2] Agholme L. Lindström T. Kågedal K. Marcusson J. Hallbeck M. (2010). An in vitro model for neuroscience: differentiation of SH-SY5Y cells into cells with morphological and biochemical characteristics of mature neurons. *J. Alzheimers. Dis.* 20 1069–1082. 10.3233/JAD-2010-091363 20413890

[B3] Asai H. Ikezu S. Tsunoda S. Medalla M. Luebke J. Haydar T. (2015). Depletion of microglia and inhibition of exosome synthesis halt tau propagation. *Nat. Neurosci.* 18 1584–1593. 10.1038/nn.4132 26436904 PMC4694577

[B4] Berning B. A. Walker A. K. (2019). The pathobiology of TDP-43 C-terminal fragments in ALS and FTLD. *Front. Neurosci.* 13:335. 10.3389/fnins.2019.00335 31031584 PMC6470282

[B5] Brettschneider J. Del Tredici K. Toledo J. B. Robinson J. L. Irwin D. J. Grossman M. (2013). Stages of pTDP-43 pathology in amyotrophic lateral sclerosis. *Ann. Neurol.* 74 20–38. 10.1002/ana.23937 23686809 PMC3785076

[B6] Chang C. K. Wu T. H. Wu C. Y. Chiang M. H. Toh E. K. W. Hsu Y. C. (2012). The N-terminus of TDP-43 promotes its oligomerization and enhances DNA binding affinity. *Biochem. Biophys. Res. Commun.* 425 219–224. 10.1016/j.bbrc.2012.07.071 22835933

[B7] Clavaguera F. Bolmont T. Crowther R. A. Abramowski D. Frank S. Probst A. (2009). Transmission and spreading of tauopathy in transgenic mouse brain. *Nat. Cell Biol.* 11 909–913. 10.1038/ncb1901 19503072 PMC2726961

[B8] Danzer K. M. Kranich L. R. Ruf W. P. Cagsal-Getkin O. Winslow A. R. Zhu L. (2012). Exosomal cell-to-cell transmission of alpha synuclein oligomers. *Mol. Neurodegener.* 7:42. 10.1186/1750-1326-7-42 22920859 PMC3483256

[B9] Desplats P. A. Lee H.-J. Bae E.-J. E.-J. Patrick C. Rockenstein E. Crews L. (2009). Inclusion formation and neuronal cell death through neuron-to-neuron transmission of a-synuclein. *Proc. Natl. Acad. Sci. U.S.A.* 106 13010–13015. 10.1073/pnas.0909073106 19651612 PMC2722313

[B10] Ding X. Ma M. Teng J. Teng R. K. F. Zhou S. Yin J. (2015). Exposure to ALS-FTD-CSF generates TDP-43 aggregates in glioblastoma cells through exosomes and TNTs-like structure. *Oncotarget* 6 24178–24191. 10.18632/oncotarget.4680 26172304 PMC4695178

[B11] Domert J. Rao S. B. Agholme L. Brorsson A.-C. Marcusson J. Hallbeck M. (2014). Spreading of amyloid-β peptides via neuritic cell-to-cell transfer is dependent on insufficient cellular clearance. *Neurobiol. Dis.* 65 82–92. 10.1016/j.nbd.2013.12.019 24412310

[B12] Domert J. Sackmann C. Agholme L. Bergström J. Ingelsson M. Hallbeck M. (2016). Aggregated alpha-synuclein transfer efficiently between cultured human neuron- like cells and localize to lysosomes. *PLoS One* 11:500. 10.1371/journal.pone.0168700 28030591 PMC5193351

[B13] Feiler M. S. Strobel B. Freischmidt A. Helferich A. M. Kappel J. Brewer B. M. (2015). TDP-43 is intercellularly transmitted across axon terminals. *J. Cell Biol.* 211 897–911. 10.1083/jcb.201504057 26598621 PMC4657165

[B14] Feneberg E. Steinacker P. Lehnert S. Schneider A. Walther P. Thal D. R. (2014). Limited role of free TDP-43 as a diagnostic tool in neurodegenerative diseases. *Amyotroph. Lateral Scler. Front. Degener.* 15 351–356. 10.3109/21678421.2014.905606 24834468

[B15] Gu J. Chen F. Iqbal K. Gong C. X. Wang X. Liu F. (2017). Transactive response DNA-binding protein 43 (TDP-43) regulates alternative splicing of tau exon 10: implications for the pathogenesis of tauopathies. *J. Biol. Chem.* 292 10600–10612. 10.1074/jbc.M117.783498 28487370 PMC5481566

[B16] Gustafsson G. Lööv C. Persson E. Lázaro D. F. Takeda S. Bergström J. (2018). Secretion and uptake of α-Synuclein via extracellular vesicles in cultured cells. *Cell. Mol. Neurobiol.* 38 1539–1550. 10.1007/s10571-018-0622-5 30288631 PMC6223723

[B17] Hanspal M. A. Dobson C. M. Yerbury J. J. Kumita J. R. (2017). The relevance of contact-independent cell-to-cell transfer of TDP-43 and SOD1 in amyotrophic lateral sclerosis. *Biochim. Biophys. Acta Mol. Basis Dis.* 1863 2762–2771. 10.1016/j.bbadis.2017.07.007 28711596 PMC6565888

[B18] Iguchi Y. Eid L. Parent M. Soucy G. Bareil C. Riku Y. (2016). Exosome secretion is a key pathway for clearance of pathological TDP-43. *Brain* 139 3187–3201. 10.1093/brain/aww237 27679482 PMC5840881

[B19] James B. D. Wilson R. S. Boyle P. A. Trojanowski J. Q. Bennett D. A. Schneider J. A. (2016). TDP-43 stage, mixed pathologies, and clinical Alzheimer’s-type dementia. *Brain* 139 2983–2993. 10.1093/brain/aww224 27694152 PMC5091047

[B20] Josephs K. A. Murray M. E. Whitwell J. L. Parisi J. E. Petrucelli L. Jack C. R. (2014a). Staging TDP-43 pathology in Alzheimer’s disease. *Acta Neuropathol.* 127 441–450. 10.1007/s00401-013-1211-9 24240737 PMC3944799

[B21] Josephs K. A. Whitwell J. L. Weigand S. D. Murray M. E. Tosakulwong N. Liesinger A. M. (2014b). TDP-43 is a key player in the clinical features associated with Alzheimer’s disease. *Acta Neuropathol.* 127 811–824. 10.1007/s00401-014-1269-z 24659241 PMC4172544

[B22] Koga S. Kouri N. Walton R. L. Ebbert M. T. W. Josephs K. A. Litvan I. (2018). Corticobasal degeneration with TDP-43 pathology presenting with progressive supranuclear palsy syndrome: a distinct clinicopathologic subtype. *Acta Neuropathol.* 136 389–404. 10.1007/s00401-018-1878-z 29926172 PMC6309287

[B23] Kraemer B. C. Schuck T. Wheeler J. M. Robinson L. C. Trojanowski J. Q. Lee V. M. Y. (2010). Loss of murine TDP-43 disrupts motor function and plays an essential role in embryogenesis. *Acta Neuropathol.* 119 409–419. 10.1007/s00401-010-0659-0 20198480 PMC2880609

[B24] Laferrière F. Maniecka Z. Pérez-Berlanga M. Hruska-Plochan M. Gilhespy L. Hock E.-M. (2019). TDP-43 extracted from frontotemporal lobar degeneration subject brains displays distinct aggregate assemblies and neurotoxic effects reflecting disease progression rates. *Nat. Neurosci.* 22 65–77. 10.1038/s41593-018-0294-y 30559480

[B25] Ling J. P. Pletnikova O. Troncoso J. C. Wong P. C. (2015). TDP-43 repression of nonconserved cryptic exons is compromised in ALS-FTD. *Science* 349 650–655. 10.1126/science.aab0983 26250685 PMC4825810

[B26] Luk K. C. Kehm V. Carroll J. Zhang B. Brien P. O. John Q. (2012). Pathological α-synuclein transmission initiates Parkinson-like Neurodegeneration in non-transgenic mice. *Science* 338 949–953. 10.1126/science.1227157.Pathological 23161999 PMC3552321

[B27] Mishima T. Koga S. Lin W.-L. Kasanuki K. Castanedes-Casey M. Wszolek Z. K. (2017). Perry syndrome: a distinctive type of TDP-43 proteinopathy. *J. Neuropathol. Exp. Neurol.* 76 676–682. 10.1093/jnen/nlx049 28789478 PMC5901076

[B28] Nath S. Agholme L. Kurudenkandy F. R. Granseth B. Marcusson J. Hallbeck M. (2012). Spreading of neurodegenerative pathology via neuron-to-neuron transmission of β-amyloid. *J. Neurosci.* 32 8767–8777. 10.1523/JNEUROSCI.0615-12.2012 22745479 PMC6622335

[B29] Neumann M. Sampathu D. M. Kwong L. K. Truax A. C. Micsenyi M. C. Chou T. T. (2006). Ubiquitinated TDP-43 in frontotemporal lobar degeneration and amyotrophic lateral sclerosis. *Science* 314 130–133. 10.1126/science.1134108 17023659

[B30] Nonaka T. Masuda-Suzukake M. Arai T. Hasegawa Y. Akatsu H. Obi T. (2013). Prion-like properties of pathological TDP-43 aggregates from diseased brains. *Cell Rep.* 4 124–134. 10.1016/j.celrep.2013.06.007 23831027

[B31] Pokrishevsky E. Grad L. I. Cashman N. R. (2016). TDP-43 or FUS-induced misfolded human wild-type SOD1 can propagate intercellularly in a prion-like fashion. *Sci. Rep.* 6 1–10. 10.1038/srep22155 26926802 PMC4772009

[B32] Porta S. Xu Y. Restrepo C. R. Kwong L. K. Zhang B. Brown H. J. (2018). Patient-derived frontotemporal lobar degeneration brain extracts induce formation and spreading of TDP-43 pathology in vivo. *Nat. Commun.* 9:4220. 10.1038/s41467-018-06548-9 30310141 PMC6181940

[B33] Rajendran L. Honsho M. Zahn T. R. Keller P. Geiger K. D. Verkade P. (2006). Alzheimer’s disease beta-amyloid peptides are released in association with exosomes. *Proc. Natl. Acad. Sci. U.S.A.* 103 11172–11177. 10.1073/pnas.0603838103 16837572 PMC1544060

[B34] Robinson J. L. Lee E. B. Xie S. X. Rennert L. Suh E. Bredenberg C. (2018). Neurodegenerative disease concomitant proteinopathies are prevalent, age-related and APOE4-associated. *Brain* 141 2181–2193. 10.1093/brain/awy146 29878075 PMC6022546

[B35] Sackmann V. Ansell A. Sackmann C. Lund H. Harris R. A. Hallbeck M. (2017). Anti-inflammatory (M2) macrophage media reduce transmission of oligomeric amyloid beta in differentiated SH-SY5Y cells. *Neurobiol. Aging* 60 173–182. 10.1016/j.neurobiolaging.2017.08.022 28969867

[B36] Sackmann V. Sardar Sinha M. Sackmann C. Civitelli L. Bergström J. Ansell-Schultz A. (2019). Inhibition of nSMase2 reduces the transfer of oligomeric α-synuclein irrespective of hypoxia. *Front. Mol. Neurosci.* 12:200. 10.3389/FNMOL.2019.00200 31555088 PMC6724746

[B37] Sardar Sinha M. Ansell-Schultz A. Civitelli L. Hildesjö C. Larsson M. Lannfelt L. (2018). Alzheimer’s disease pathology propagation by exosomes containing toxic amyloid-beta oligomers. *Acta Neuropathol.* 136 41–56. 10.1007/s00401-018-1868-1 29934873 PMC6015111

[B38] Sephton C. F. Good S. K. Atkin S. Dewey C. M. Mayer P. Herz J. (2010). TDP-43 Is a developmentally regulated protein essential for early embryonic development. *J. Biol. Chem.* 285 6826–6834. 10.1074/jbc.M109.061846 20040602 PMC2825476

[B39] Shiina Y. Arima K. Tabunoki H. Satoh J. (2010). TDP-43 dimerizes in human cells in culture. *Cell. Mol. Neurobiol.* 30 641–652. 10.1007/s10571-009-9489-9 20043239 PMC11498789

[B40] Shimonaka S. Nonaka T. Suzuki G. Hisanaga S. I. Hasegawa M. (2016). Templated aggregation of TAR DNA-binding protein of 43 kDa (TDP-43) by seeding with TDP-43 peptide fibrils. *J. Biol. Chem.* 291 8896–8907. 10.1074/jbc.M115.713552 26887947 PMC4861459

[B41] Smethurst P. Newcombe J. Troakes C. Simone R. Chen Y. R. Patani R. (2016). In vitro prion-like behaviour of TDP-43 in ALS. *Neurobiol. Dis.* 96 236–247. 10.1016/j.nbd.2016.08.007 27590623 PMC5113659

[B42] Sun M. Bell W. LaClair K. D. Ling J. P. Han H. Kageyama Y. (2017). Cryptic exon incorporation occurs in Alzheimer’s brain lacking TDP-43 inclusion but exhibiting nuclear clearance of TDP-43. *Acta Neuropathol.* 133 923–931. 10.1007/s00401-017-1701-2 28332094 PMC5444385

[B43] Wu L.-S. Cheng W.-C. Hou S.-C. Yan Y.-T. Jiang S.-T. Shen C.-K. J. (2010). TDP-43, a neuro-pathosignature factor, is essential for early mouse embryogenesis. *Genesis* 48 56–62. 10.1002/dvg.20584 20014337

[B44] Yang C. Tan W. Whittle C. Qiu L. Cao L. Akbarian S. (2010). The C-terminal TDP-43 fragments have a high aggregation propensity and harm neurons by a dominant-negative mechanism. *PLoS One* 5:e15878. 10.1371/journal.pone.0015878 21209826 PMC3013128

[B45] Zacco E. Martin S. R. Thorogate R. Pastore A. (2018). The RNA-recognition motifs of TAR DNA-binding protein 43 may play a role in the aberrant self-assembly of the protein. *Front. Mol. Neurosci.* 11:372. 10.3389/fnmol.2018.00372 30356856 PMC6190850

[B46] Zeineddine R. Whiten D. R. Farrawell N. E. McAlary L. Hanspal M. A. Kumita J. R. (2017). Flow cytometric measurement of the cellular propagation of TDP-43 aggregation. *Prion* 11 195–204. 10.1080/19336896.2017.1314426 28486039 PMC5480386

[B47] Zhang Y. J. Caulfield T. Xu Y. F. Gendron T. F. Hubbard J. Stetler C. (2013). The dual functions of the extreme N-terminus of TDP-43 in regulating its biological activity and inclusion formation. *Hum. Mol. Genet.* 22 3112–3122. 10.1093/hmg/ddt166 23575225 PMC3699067

[B48] Zhang Y.-J. Xu Y.-F. Cook C. Gendron T. F. Roettges P. Link C. D. (2009). Aberrant cleavage of TDP-43 enhances aggregation and cellular toxicity. *Proc. Natl. Acad. Sci. U.S.A.* 106 7607–7612. 10.1073/pnas.0900688106 19383787 PMC2671323

