# Supplementary figures and images for: TDP-43 Is Efficiently Transferred Between Neuron-Like Cells in a Manner Enhanced by Preservation of Its N-Terminus but Independent of Extracellular Vesicles
Source: Front Neurosci. 2020 Jun 11;14:540. doi: 10.3389/fnins.2020.00540 (PMC7301158; doi:10.3389/fnins.2020.00540)

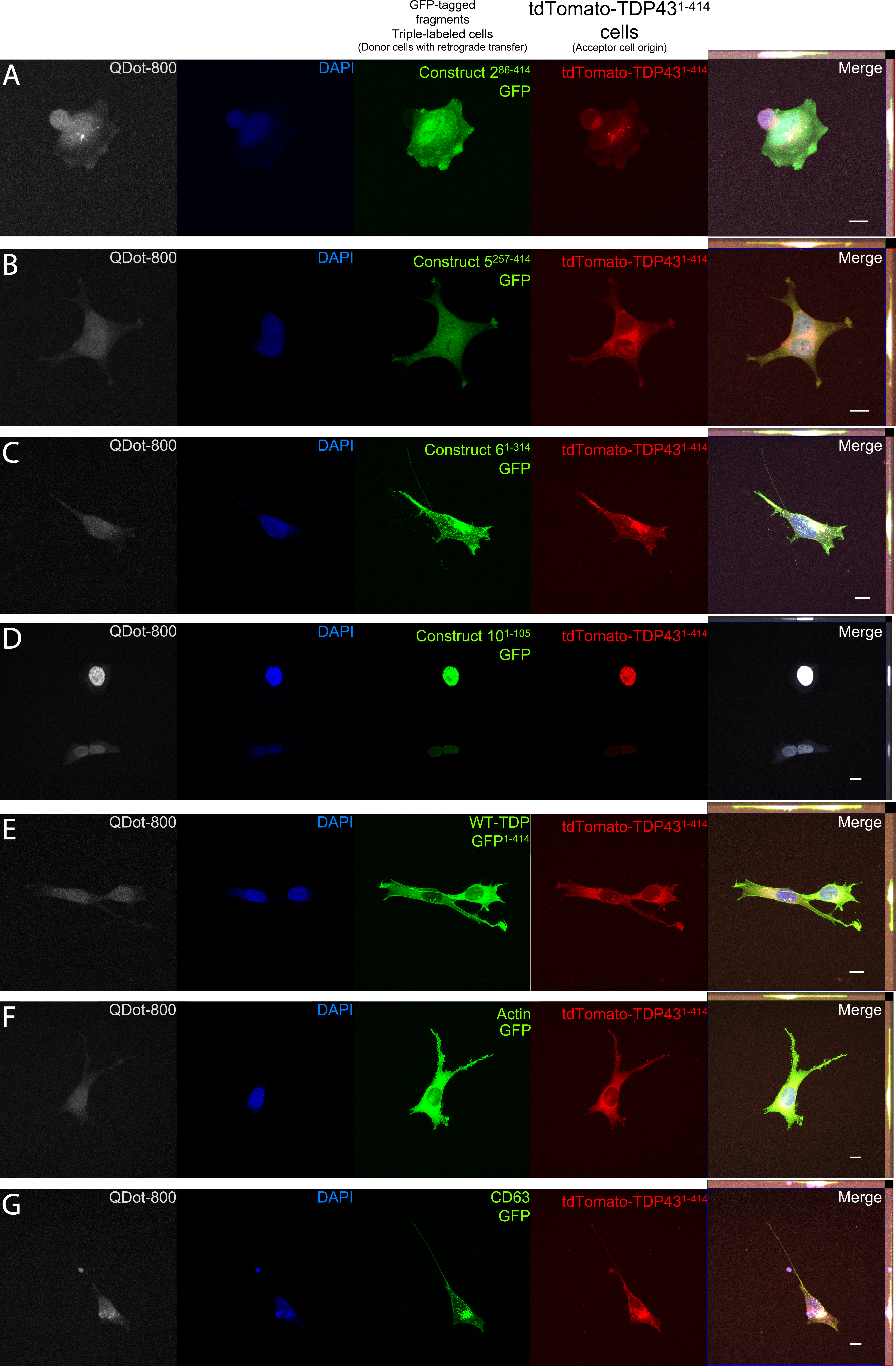

Supplement: FIGURE S4 — Representative images depicting subcellular localization of TDP-43 in donor cells exhibiting retrograde transfer. GFP-tagged protein expressing donor cells [+GFP (green), +Qdot-800 (white), +td-tomatoTDP431– 414 (red)] display triple labeling after retrograde transfer of td-tomatoTDP431– 414 originating from acceptor cells. Acceptor cells (td-tomatoTDP431– 414) transferred proteins to the donor cells (+GFP and +Qdot-800; retrograde transfer), albeit less efficiently than donor to acceptor cells (anterograde; see Figure 3B). Cells which were triple-positive (+GFP, +Qdot-800, and +td-tomatoTDP431– 414) were selected using FACS and fixed for confocal microscopy. This data indicates that full-length TDP-43 and truncated TDP-43 both harbor the ability to transfer between cells. Subcellular localization of TDP-43 fragments (A–D), full-length TDP-43 (E), actin (F) and CD63 (G) are akin to those observed in previous experiments (see Figure 2 and Supplementary Figures S3, S5). The final column contains Z-projections. Scale bar = 10 μm. [file Image_4.JPEG]

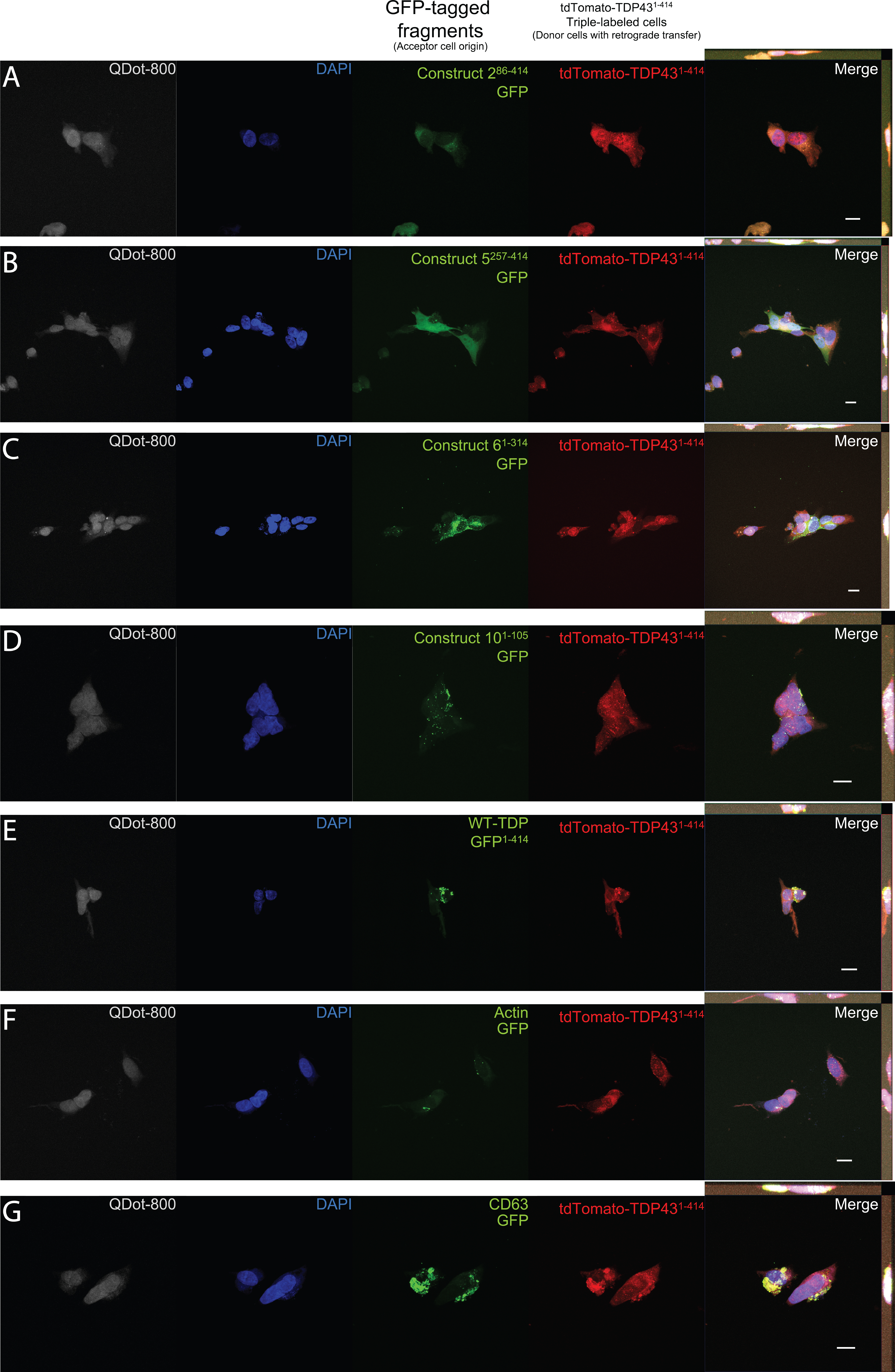

Supplement: FIGURE S5 — Representative images depicting subcellular localization of TDP-43 fragments in donor exhibiting retrograde transfer. td-tomatoTDP431– 414 expressing donor cells [+td-tomatoTDP431– 414 (red), +Qdot-800 (white), and +GFP (green)] display triple labeling after retrograde transfer of GFP-tagged TDP-43 truncated proteins originating from acceptor cells. Acceptor cells (GFP) transferred proteins to the donor cells (+td-tomatoTDP431– 414 and +Qdot-800; retrograde transfer), albeit less efficiently than donor to acceptor (see Figures 3B,C). Cells which were triple-positive (+GFP, +td-tomatoTDP431– 414, and +Qdot800) were selected using FACS and fixed for confocal microscopy. This data indicates that most of the protein transfer occurs from donor to acceptor cell (anterograde), however, there is a modest amount of protein transfer from the acceptor cells to the donor cells (retrograde). Subcellular localization of TDP-43 fragments (A–D), full-length TDP-43 (E), actin (F) and CD63 (G) are akin to those observed in previous experiments. The final column contains Z-projections. Scale bar = 10 μm. [file Image_5.JPEG]

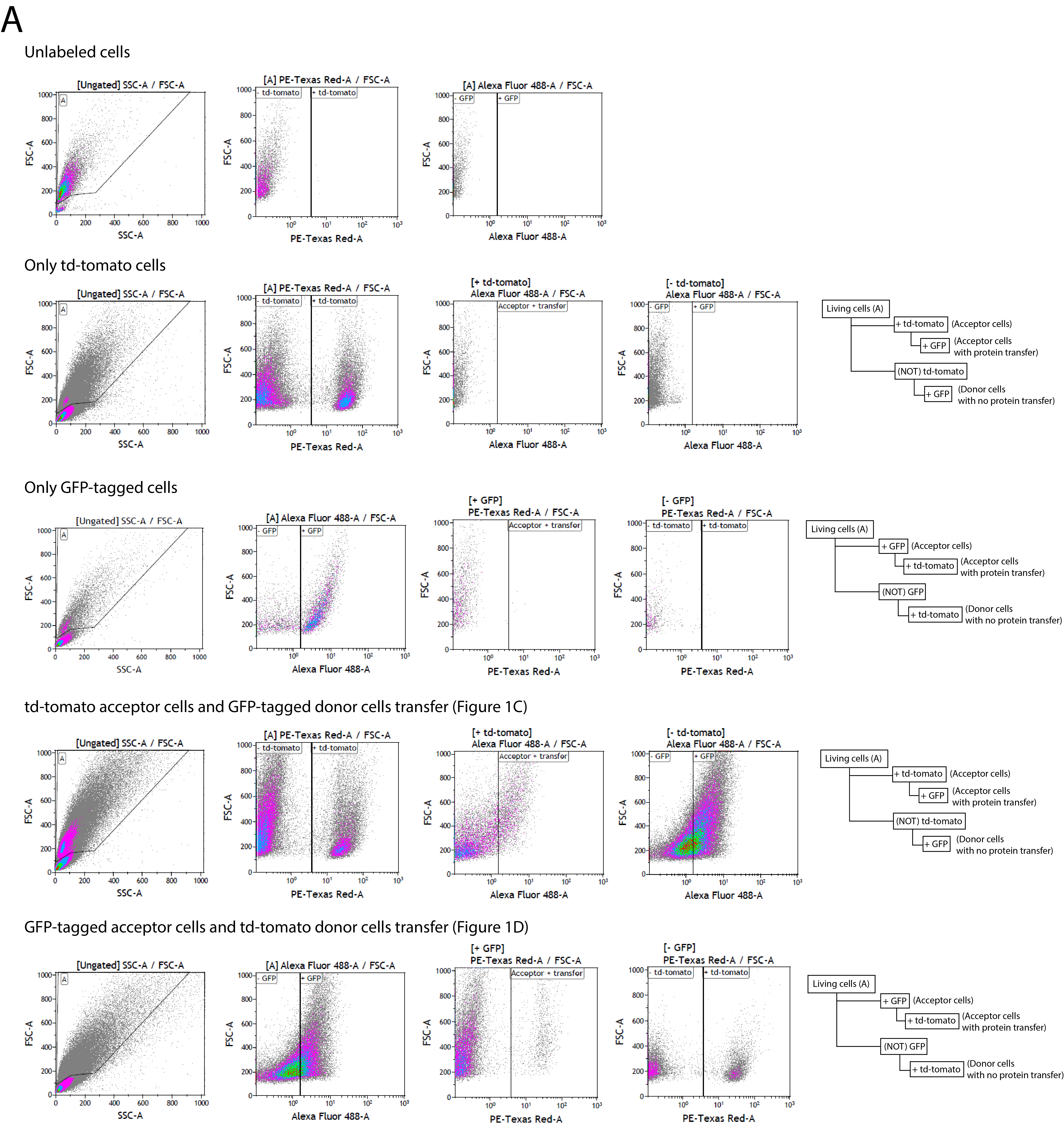

Supplement: FIGURE S6 — Gating strategies for cell sorting by FACS and representative population graphs. Unlabeled cells were first used to determine the live cell population and td-tomato (PE-Texas Red) and GFP (Alexa Fluor 488) negative cells using cut-off values determined by the authors. This was followed by gating strategies using only td-tomato or GFP-tagged acceptor cells. (A) Gating strategies for Figures 1C,D and (B) for Figures 4B–D. (C) Figures 3B,C utilized Qdot labeled donor cells that were first gated and then followed the gating strategies as the previous sets. Acceptor cells with protein transfer were then used to determine the percentage of transfer and sorted for confocal microscopy. [file Image_6.TIF]

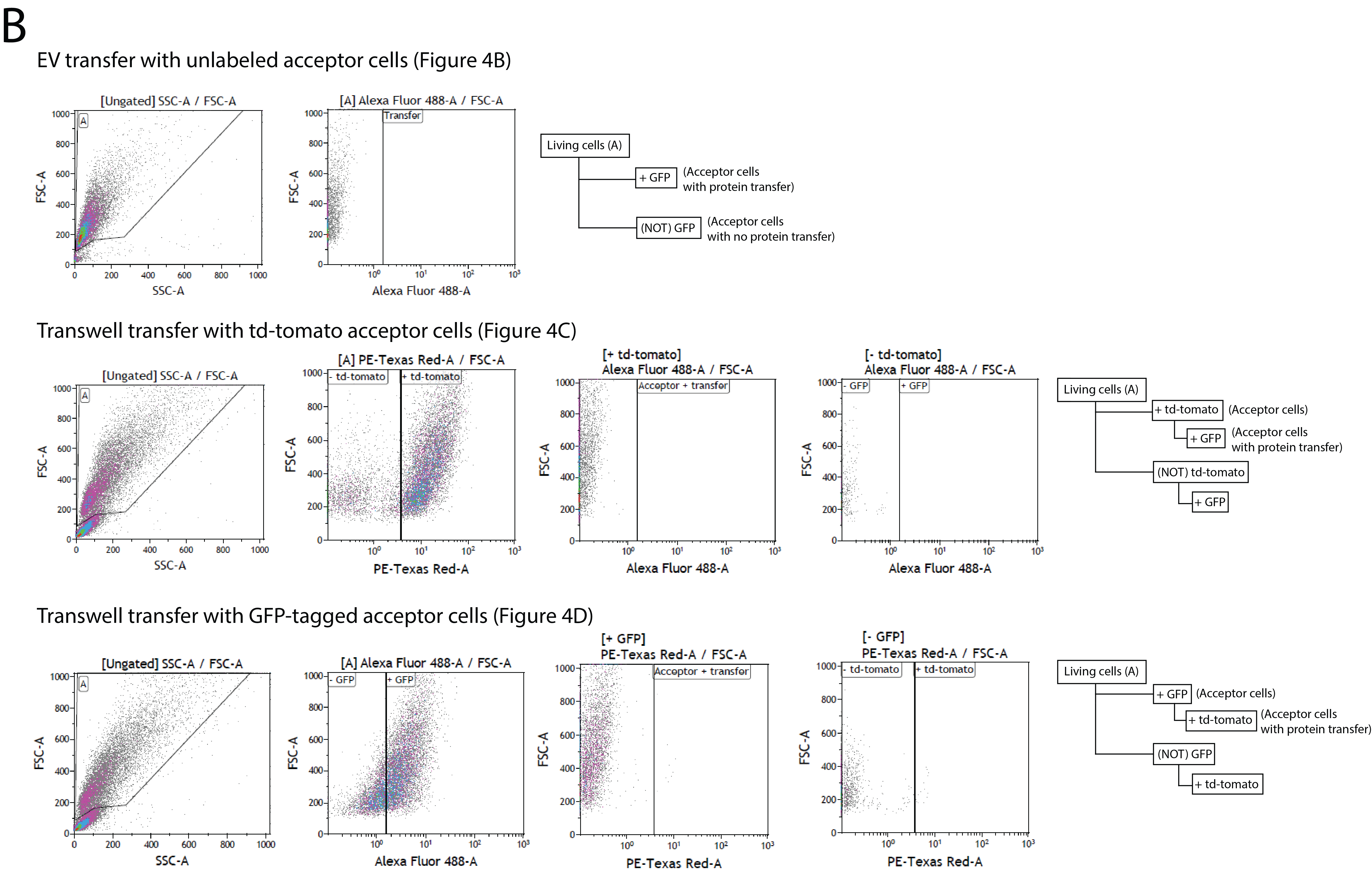

Supplement: Supplementary file 7 [file Image_7.TIF]

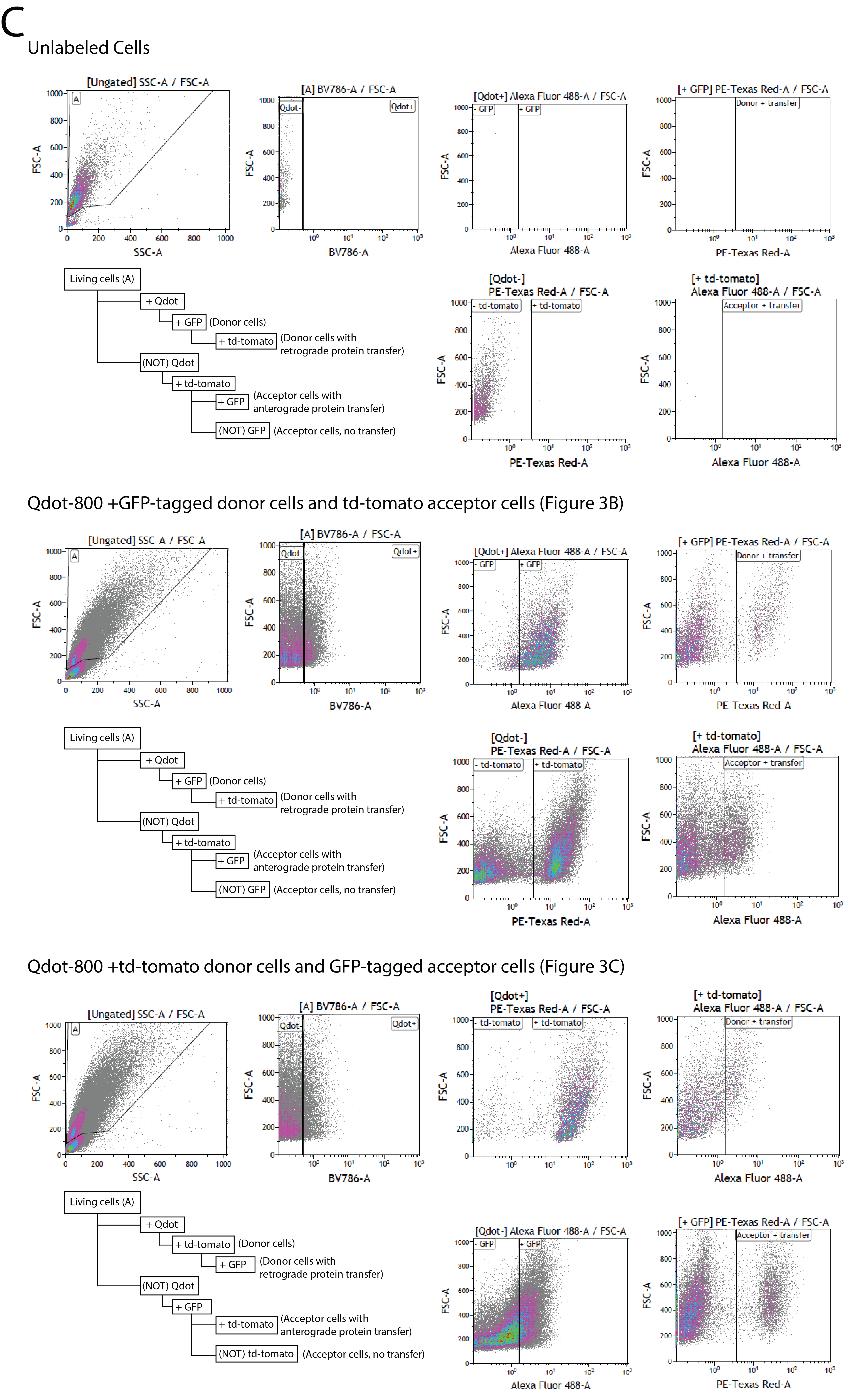

Supplement: Supplementary file 8 [file Image_8.TIF]
