# Supplementary material for: TDP-43 Is Efficiently Transferred Between Neuron-Like Cells in a Manner Enhanced by Preservation of Its N-Terminus but Independent of Extracellular Vesicles
Source: Front Neurosci. 2020 Jun 11;14:540. doi: 10.3389/fnins.2020.00540 (PMC7301158; doi:10.3389/fnins.2020.00540)
Supplement: TABLE S1 — List of plasmids used in the study. [file Table_1.DOCX]

Supplementary Table S1. List of plasmids used in the study.

| Plasmid | Fluorescent protein | TDP-43 Sequence | Distributor |
| --- | --- | --- | --- |
| Construct 2^86-414^ | EGFP (C-terminus) | 86-414 | Zuoshang Xu (Addgene plasmid # 28195) |
| Construct 3^170-414^ | EGFP (C-terminus) | 170-414 | Zuoshang Xu (Addgene plasmid # 28196) |
| Construct 4^216-414^ | EGFP (C-terminus) | 216-414 | Zuoshang Xu (Addgene plasmid # 28197) |
| Construct 5^257-414^ | EGFP (C-terminus) | 257-414 | Zuoshang Xu (Addgene plasmid # 28198) |
| Construct 6^1-314^ | EGFP (C-terminus) | 1-314 | Zuoshang Xu (Addgene plasmid # 28199) |
| Construct 10^1-105^ | EGFP (C-terminus) | 1-105 | Zuoshang Xu (Addgene plasmid # 28195) |
| WT-TDP-43^1-414^ | TurboGFP (C-terminus) | 1-414 | Origene RG210639 |
| td-tomatoTDP43^1-414^ | td-tomato (C-terminus) | 1-414 | Zuoshang Xu (Addgene plasmid # 28205) |
| AA55-414 | TurboGFP (C-terminus) | 55-414 | Modified from Origene RG210639 |
| CD63 GFP | EGFP (N-terminus) | N/A | Paul Luzio (Addgene plasmid # 62964) |
| Actin GFP | mEGFP (N-terminus) | N/A | Ryohei Yasuda (Addgene plasmid # 21948) |
| pcDNA GFP | EGFP (N/A) | N/A | Doug Golenbock (Addgene plasmid # 13031) |

Supplementary Table S2. List of antibodies used in the study.

| Antibody | Dilution | Distributor | Product Number |
| --- | --- | --- | --- |
| Flotillin-1 | 1:1000 | BD Transduction Laboratories | 610820 |
| TDP-43 (N-terminus) | 1:1000 | Aviva Systems Biology | OAAB10063 |
| TDP-43 (C-terminus) | 1:1000 | Biolegend | 808301 (Clone TDP2H4) |
| pTDP-43 (pSer409/410) | 1:1000 | Biolegend | 829901 (Clone 1D3/TDP-43) |
| GFP | 1:1000 | Invitrogen | A11122 |
| Goat anti-mouse-HRP | 1:2000 | Dako | P0447 |
| Goat anti-rabbit-HRP | 1:2000 | Dako | P0448 |
| Rabbit anti-rat-HRP | 1:2000 | Dako | P0450 |
| GAPDH-HRP | 1:1000 | Novus Biologicals | NB300-328H (Clone 2D4A7) |

Supplementary Table S3. ANOVA table corresponding to Figure 1C. The mean values provided here are the percentage of td-tomatoTDP43^1-414^ acceptor cells that are double labeled (acceptor cells with positive transfer).

| **Test details** | **Mean 1** | **Mean 2** | **Mean Diff.** | **SE of diff.** | **n1** | **n2** | **95% CI of diff.** | **Summary** |
| --- | --- | --- | --- | --- | --- | --- | --- | --- |
| Construct 2^86-414^ vs. Construct 5^257-414^ | 7.256 | 7.728 | -0.4722 | 0.9188 | 7 | 7 | -3.313 to 2.369 | ns |
| Construct 2^86-414^ vs. Construct 6^1-314^ | 7.256 | 12.95 | -5.691 | 0.9188 | 7 | 7 | -8.532 to -2.850 | **** |
| Construct 2^86-414^ vs. Construct 10^1-105^ | 7.256 | 6.958 | 0.2983 | 0.9563 | 7 | 6 | -2.659 to 3.255 | ns |
| Construct 2^86-414^ vs. WT-TDP-GFP^1-414^ | 7.256 | 8.503 | -1.247 | 0.8471 | 7 | 10 | -3.866 to 1.372 | ns |
| Construct 2^86-414^ vs. Actin GFP | 7.256 | 5.821 | 1.435 | 0.9563 | 7 | 6 | -1.522 to 4.392 | ns |
| Construct 2^86-414^ vs. CD63 GFP | 7.256 | 15.15 | -7.898 | 0.9188 | 7 | 7 | -10.74 to -5.058 | **** |
| Construct 5^257-414^ vs. Construct 6^1-314^ | 7.728 | 12.95 | -5.218 | 0.9188 | 7 | 7 | -8.059 to -2.378 | **** |
| Construct 5^257-414^ vs. Construct 10^1-105^ | 7.728 | 6.958 | 0.7705 | 0.9563 | 7 | 6 | -2.186 to 3.727 | ns |
| Construct 5^257-414^ vs. WT-TDP-GFP^1-414^ | 7.728 | 8.503 | -0.7751 | 0.8471 | 7 | 10 | -3.394 to 1.844 | ns |
| Construct 5^257-414^ vs. Actin GFP | 7.728 | 5.821 | 1.907 | 0.9563 | 7 | 6 | -1.050 to 4.864 | ns |
| Construct 5^257-414^ vs. CD63 GFP | 7.728 | 15.15 | -7.426 | 0.9188 | 7 | 7 | -10.27 to -4.585 | **** |
| Construct 6^1-314^ vs. Construct 10^1-105^ | 12.95 | 6.958 | 5.989 | 0.9563 | 7 | 6 | 3.032 to 8.946 | **** |
| Construct 6^1-314^ vs. WT-TDP-GFP^1-414^ | 12.95 | 8.503 | 4.443 | 0.8471 | 7 | 10 | 1.824 to 7.063 | **** |
| Construct 6^1-314^ vs. Actin GFP | 12.95 | 5.821 | 7.126 | 0.9563 | 7 | 6 | 4.169 to 10.08 | **** |
| Construct 6^1-314^ vs. CD63 GFP | 12.95 | 15.15 | -2.208 | 0.9188 | 7 | 7 | -5.049 to 0.6331 | ns |
| Construct 10^1-105^ vs. WT-TDP-GFP^1-414^ | 6.958 | 8.503 | -1.546 | 0.8876 | 6 | 10 | -4.290 to 1.199 | ns |
| Construct 10^1-105^ vs. Actin GFP | 6.958 | 5.821 | 1.137 | 0.9924 | 6 | 6 | -1.932 to 4.205 | ns |
| Construct 10^1-105^ vs. CD63 GFP | 6.958 | 15.15 | -8.197 | 0.9563 | 6 | 7 | -11.15 to -5.240 | **** |
| WT-TDP-GFP^1-414^ vs. Actin GFP | 8.503 | 5.821 | 2.682 | 0.8876 | 10 | 6 | -0.06244 to 5.427 | ns |
| WT-TDP-GFP^1-414^ vs. CD63 GFP | 8.503 | 15.15 | -6.651 | 0.8471 | 10 | 7 | -9.270 to -4.032 | **** |
| Actin GFP vs. CD63 GFP | 5.821 | 15.15 | -9.333 | 0.9563 | 6 | 7 | -12.29 to -6.376 | **** |

Supplementary Table S4. ANOVA table corresponding to Figure 1D. The mean values provided here are the percentage of GFP-tagged protein expressing acceptor cells that are double labeled (acceptor cells with positive transfer).

| **Test details** | **Mean 1** | **Mean 2** | **Mean Diff.** | **SE of diff.** | **n1** | **n2** | **95% CI of diff.** | **Summary** |
| --- | --- | --- | --- | --- | --- | --- | --- | --- |
| Construct 2^86-414^ vs. Construct 5^257-414^ | 4.948 | 4.225 | 0.7226 | 0.6804 | 6 | 7 | -1.412 to 2.857 | ns |
| Construct 2^86-414^ vs. Construct 6^1-314^ | 4.948 | 7.864 | -2.916 | 0.7061 | 6 | 6 | -5.131 to -0.7008 | ** |
| Construct 2^86-414^ vs. Construct 10^1-105^ | 4.948 | 2.947 | 2.001 | 0.7894 | 6 | 4 | -0.4751 to 4.478 | ns |
| Construct 2^86-414^ vs. WT-TDP-GFP^1-414^ | 4.948 | 11.07 | -6.121 | 0.7061 | 6 | 6 | -8.336 to -3.906 | **** |
| Construct 2^86-414^ vs. Actin GFP | 4.948 | 7.725 | -2.777 | 0.7894 | 6 | 4 | -5.254 to -0.3007 | * |
| Construct 2^86-414^ vs. CD63 GFP | 4.948 | 9.107 | -4.159 | 0.6804 | 6 | 7 | -6.294 to -2.025 | **** |
| Construct 5^257-414^ vs. Construct 6^1-314^ | 4.225 | 7.864 | -3.638 | 0.6804 | 7 | 6 | -5.773 to -1.504 | *** |
| Construct 5^257-414^ vs. Construct 10^1-105^ | 4.225 | 2.947 | 1.279 | 0.7666 | 7 | 4 | -1.126 to 3.684 | ns |
| Construct 5^257-414^ vs. WT-TDP-GFP^1-414^ | 4.225 | 11.07 | -6.844 | 0.6804 | 7 | 6 | -8.978 to -4.709 | **** |
| Construct 5^257-414^ vs. Actin GFP | 4.225 | 7.725 | -3.5 | 0.7666 | 7 | 4 | -5.905 to -1.095 | ** |
| Construct 5^257-414^ vs. CD63 GFP | 4.225 | 9.107 | -4.882 | 0.6537 | 7 | 7 | -6.933 to -2.831 | **** |
| Construct 6^1-314^ vs. Construct 10^1-105^ | 7.864 | 2.947 | 4.917 | 0.7894 | 6 | 4 | 2.441 to 7.394 | **** |
| Construct 6^1-314^ vs. WT-TDP-GFP^1-414^ | 7.864 | 11.07 | -3.205 | 0.7061 | 6 | 6 | -5.420 to -0.9902 | ** |
| Construct 6^1-314^ vs. Actin GFP | 7.864 | 7.725 | 0.1386 | 0.7894 | 6 | 4 | -2.338 to 2.615 | ns |
| Construct 6^1-314^ vs. CD63 GFP | 7.864 | 9.107 | -1.244 | 0.6804 | 6 | 7 | -3.378 to 0.8909 | ns |
| Construct 10^1-105^ vs. WT-TDP-GFP^1-414^ | 2.947 | 11.07 | -8.123 | 0.7894 | 4 | 6 | -10.60 to -5.646 | **** |
| Construct 10^1-105^ vs. Actin GFP | 2.947 | 7.725 | -4.779 | 0.8648 | 4 | 4 | -7.491 to -2.066 | **** |
| Construct 10^1-105^ vs. CD63 GFP | 2.947 | 9.107 | -6.161 | 0.7666 | 4 | 7 | -8.566 to -3.756 | **** |
| WT-TDP-GFP^1-414^ vs. Actin GFP | 11.07 | 7.725 | 3.344 | 0.7894 | 6 | 4 | 0.8674 to 5.820 | ** |
| WT-TDP-GFP^1-414^ vs. CD63 GFP | 11.07 | 9.107 | 1.962 | 0.6804 | 6 | 7 | -0.1728 to 4.096 | ns |
| Actin GFP vs. CD63 GFP | 7.725 | 9.107 | -1.382 | 0.7666 | 4 | 7 | -3.787 to 1.023 | ns |

Supplementary Table S5. ANOVA table corresponding to Figure 3B. The mean values provided here are: of the total transfer events measured, those that were anterograde (td-tomatoTDP43^1-414^ acceptor cells that are double labeled). The retrograde values are the difference, out of 100, and therefore give the same, but inverse, tabular results.

| **Test details** | **Mean 1** | **Mean 2** | **Mean Diff.** | **SE of diff.** | **n1** | **n2** | **95% CI of diff.** | **Summary** |
| --- | --- | --- | --- | --- | --- | --- | --- | --- |
| Construct 2^86-414^ vs. Construct 5^257-414^ | 76.31 | 91.83 | -15.52 | 0.8037 | 4 | 3 | -18.19 to -12.84 | **** |
| Construct 2^86-414^ vs. Construct 6^1-314^ | 76.31 | 92.62 | -16.3 | 0.8037 | 4 | 3 | -18.98 to -13.63 | **** |
| Construct 2^86-414^ vs. Construct 10^1-105^ | 76.31 | 84.12 | -7.812 | 0.7441 | 4 | 4 | -10.29 to -5.337 | **** |
| Construct 2^86-414^ vs. WT-TDP-GFP^1-414^ | 76.31 | 90.02 | -13.71 | 0.7441 | 4 | 4 | -16.19 to -11.23 | **** |
| Construct 2^86-414^ vs. Actin GFP | 76.31 | 91.6 | -15.29 | 0.8037 | 4 | 3 | -17.97 to -12.62 | **** |
| Construct 2^86-414^ vs. CD63 GFP | 76.31 | 87.15 | -10.83 | 0.8037 | 4 | 3 | -13.51 to -8.161 | **** |
| Construct 5^257-414^ vs. Construct 6^1-314^ | 91.83 | 92.62 | -0.7855 | 0.8592 | 3 | 3 | -3.644 to 2.073 | ns |
| Construct 5^257-414^ vs. Construct 10^1-105^ | 91.83 | 84.12 | 7.706 | 0.8037 | 3 | 4 | 5.032 to 10.38 | **** |
| Construct 5^257-414^ vs. WT-TDP-GFP^1-414^ | 91.83 | 90.02 | 1.808 | 0.8037 | 3 | 4 | -0.8660 to 4.481 | ns |
| Construct 5^257-414^ vs. Actin GFP | 91.83 | 91.6 | 0.2265 | 0.8592 | 3 | 3 | -2.632 to 3.085 | ns |
| Construct 5^257-414^ vs. CD63 GFP | 91.83 | 87.15 | 4.683 | 0.8592 | 3 | 3 | 1.825 to 7.542 | *** |
| Construct 6^1-314^ vs. Construct 10^1-105^ | 92.62 | 84.12 | 8.491 | 0.8037 | 3 | 4 | 5.817 to 11.16 | **** |
| Construct 6^1-314^ vs. WT-TDP-GFP^1-414^ | 92.62 | 90.02 | 2.593 | 0.8037 | 3 | 4 | -0.08050 to 5.267 | ns |
| Construct 6^1-314^ vs. Actin GFP | 92.62 | 91.6 | 1.012 | 0.8592 | 3 | 3 | -1.846 to 3.870 | ns |
| Construct 6^1-314^ vs. CD63 GFP | 92.62 | 87.15 | 5.469 | 0.8592 | 3 | 3 | 2.611 to 8.327 | *** |
| Construct 10^1-105^ vs. WT-TDP-GFP^1-414^ | 84.12 | 90.02 | -5.898 | 0.7441 | 4 | 4 | -8.373 to -3.422 | **** |
| Construct 10^1-105^ vs. Actin GFP | 84.12 | 91.6 | -7.479 | 0.8037 | 4 | 3 | -10.15 to -4.805 | **** |
| Construct 10^1-105^ vs. CD63 GFP | 84.12 | 87.15 | -3.022 | 0.8037 | 4 | 3 | -5.696 to -0.3485 | * |
| WT-TDP-GFP^1-414^ vs. Actin GFP | 90.02 | 91.6 | -1.581 | 0.8037 | 4 | 3 | -4.255 to 1.092 | ns |
| WT-TDP-GFP^1-414^ vs. CD63 GFP | 90.02 | 87.15 | 2.876 | 0.8037 | 4 | 3 | 0.2019 to 5.549 | * |
| Actin GFP vs. CD63 GFP | 91.6 | 87.15 | 4.457 | 0.8592 | 3 | 3 | 1.599 to 7.315 | ** |

Supplementary Table S6. ANOVA table corresponding to Figure 3C. The mean values provided here are: of the total transfer events measured, those that were anterograde (GFP-tagged protein acceptor cells that are double labeled). The retrograde values are the difference, out of 100, and therefore give the same, but inverse, tabular results.

| **Test details** | **Mean 1** | **Mean 2** | **Mean Diff.** | **SE of diff.** | **n1** | **n2** | **95% CI of diff.** | **Summary** |
| --- | --- | --- | --- | --- | --- | --- | --- | --- |
| Construct 2^86-414^ vs. Construct 5^257-414^ | 86.93 | 89.19 | -2.259 | 0.9935 | 3 | 3 | -5.651 to 1.133 | ns |
| Construct 2^86-414^ vs. Construct 6^1-314^ | 86.93 | 86.46 | 0.4636 | 0.9935 | 3 | 3 | -2.929 to 3.856 | ns |
| Construct 2^86-414^ vs. Construct 10^1-105^ | 86.93 | 73.06 | 13.87 | 0.9935 | 3 | 3 | 10.48 to 17.26 | **** |
| Construct 2^86-414^ vs. WT-TDP-GFP^1-414^ | 86.93 | 87.86 | -0.9353 | 0.9935 | 3 | 3 | -4.328 to 2.457 | ns |
| Construct 2^86-414^ vs. Actin GFP | 86.93 | 84.24 | 2.689 | 0.9935 | 3 | 3 | -0.7031 to 6.082 | ns |
| Construct 2^86-414^ vs. CD63 GFP | 86.93 | 87.27 | -0.3415 | 0.9935 | 3 | 3 | -3.734 to 3.051 | ns |
| Construct 5^257-414^ vs. Construct 6^1-314^ | 89.19 | 86.46 | 2.723 | 0.9935 | 3 | 3 | -0.6698 to 6.115 | ns |
| Construct 5^257-414^ vs. Construct 10^1-105^ | 89.19 | 73.06 | 16.13 | 0.9935 | 3 | 3 | 12.74 to 19.52 | **** |
| Construct 5^257-414^ vs. WT-TDP-GFP^1-414^ | 89.19 | 87.86 | 1.324 | 0.9935 | 3 | 3 | -2.069 to 4.716 | ns |
| Construct 5^257-414^ vs. Actin GFP | 89.19 | 84.24 | 4.948 | 0.9935 | 3 | 3 | 1.556 to 8.341 | ** |
| Construct 5^257-414^ vs. CD63 GFP | 89.19 | 87.27 | 1.918 | 0.9935 | 3 | 3 | -1.475 to 5.310 | ns |
| Construct 6^1-314^ vs. Construct 10^1-105^ | 86.46 | 73.06 | 13.41 | 0.9935 | 3 | 3 | 10.01 to 16.80 | **** |
| Construct 6^1-314^ vs. WT-TDP-GFP^1-414^ | 86.46 | 87.86 | -1.399 | 0.9935 | 3 | 3 | -4.791 to 1.993 | ns |
| Construct 6^1-314^ vs. Actin GFP | 86.46 | 84.24 | 2.226 | 0.9935 | 3 | 3 | -1.167 to 5.618 | ns |
| Construct 6^1-314^ vs. CD63 GFP | 86.46 | 87.27 | -0.805 | 0.9935 | 3 | 3 | -4.197 to 2.587 | ns |
| Construct 10^1-105^ vs. WT-TDP-GFP^1-414^ | 73.06 | 87.86 | -14.8 | 0.9935 | 3 | 3 | -18.20 to -11.41 | **** |
| Construct 10^1-105^ vs. Actin GFP | 73.06 | 84.24 | -11.18 | 0.9935 | 3 | 3 | -14.57 to -7.788 | **** |
| Construct 10^1-105^ vs. CD63 GFP | 73.06 | 87.27 | -14.21 | 0.9935 | 3 | 3 | -17.60 to -10.82 | **** |
| WT-TDP-GFP^1-414^ vs. Actin GFP | 87.86 | 84.24 | 3.625 | 0.9935 | 3 | 3 | 0.2322 to 7.017 | * |
| WT-TDP-GFP^1-414^ vs. CD63 GFP | 87.86 | 87.27 | 0.5938 | 0.9935 | 3 | 3 | -2.799 to 3.986 | ns |
| Actin GFP vs. CD63 GFP | 84.24 | 87.27 | -3.031 | 0.9935 | 3 | 3 | -6.423 to 0.3616 | ns |

Supplementary Table S7. ANOVA table corresponding to Figure 4B. The mean values provided here are the percentage of WT SH-SY5Y acceptor cells that are fluorescently labeled.

| **Test details** | **Mean 1** | **Mean 2** | **Mean Diff.** | **SE of diff.** | **n1** | **n2** | **95% CI of diff.** | **Summary** |
| --- | --- | --- | --- | --- | --- | --- | --- | --- |
| Construct 2^86-414^ vs. Construct 5^257-414^ | 0.0971 | 0.1492 | -0.0521 | 0.07508 | 7 | 6 | -0.2988 to 0.1946 | ns |
| Construct 2^86-414^ vs. Construct 6^1-314^ | 0.0971 | 0.1087 | -0.01162 | 0.07213 | 7 | 7 | -0.2486 to 0.2254 | ns |
| Construct 2^86-414^ vs. Construct 10^1-105^ | 0.0971 | 0.09503 | 0.002076 | 0.07213 | 7 | 7 | -0.2349 to 0.2391 | ns |
| Construct 2^86-414^ vs. WT-TDP-GFP^1-414^ | 0.0971 | 0.09588 | 0.001226 | 0.07213 | 7 | 7 | -0.2358 to 0.2382 | ns |
| Construct 2^86-414^ vs. td-tomatoTDP43^1-414^ | 0.0971 | 0.2183 | -0.1212 | 0.06801 | 7 | 9 | -0.3447 to 0.1022 | ns |
| Construct 2^86-414^ vs. Actin GFP | 0.0971 | 0.09233 | 0.004774 | 0.07508 | 7 | 6 | -0.2419 to 0.2514 | ns |
| Construct 2^86-414^ vs. CD63 GFP | 0.0971 | 0.07015 | 0.02695 | 0.07508 | 7 | 6 | -0.2197 to 0.2736 | ns |
| Construct 2^86-414^ vs. pcDNA GFP | 0.0971 | 0.1752 | -0.0781 | 0.07508 | 7 | 6 | -0.3248 to 0.1686 | ns |
| Construct 2^86-414^ vs. WT Unlabeled | 0.0971 | 0.1005 | -0.003406 | 0.06801 | 7 | 9 | -0.2268 to 0.2200 | ns |
| Construct 5^257-414^ vs. Construct 6^1-314^ | 0.1492 | 0.1087 | 0.04048 | 0.07508 | 6 | 7 | -0.2062 to 0.2871 | ns |
| Construct 5^257-414^ vs. Construct 10^1-105^ | 0.1492 | 0.09503 | 0.05418 | 0.07508 | 6 | 7 | -0.1925 to 0.3008 | ns |
| Construct 5^257-414^ vs. WT-TDP-GFP^1-414^ | 0.1492 | 0.09588 | 0.05333 | 0.07508 | 6 | 7 | -0.1933 to 0.3000 | ns |
| Construct 5^257-414^ vs. td-tomatoTDP43^1-414^ | 0.1492 | 0.2183 | -0.06913 | 0.07112 | 6 | 9 | -0.3028 to 0.1645 | ns |
| Construct 5^257-414^ vs. Actin GFP | 0.1492 | 0.09233 | 0.05688 | 0.07791 | 6 | 6 | -0.1991 to 0.3129 | ns |
| Construct 5^257-414^ vs. CD63 GFP | 0.1492 | 0.07015 | 0.07906 | 0.07791 | 6 | 6 | -0.1769 to 0.3350 | ns |
| Construct 5^257-414^ vs. pcDNA GFP | 0.1492 | 0.1752 | -0.026 | 0.07791 | 6 | 6 | -0.2820 to 0.2300 | ns |
| Construct 5^257-414^ vs. WT Unlabeled | 0.1492 | 0.1005 | 0.0487 | 0.07112 | 6 | 9 | -0.1850 to 0.2824 | ns |
| Construct 6^1-314^ vs. Construct 10^1-105^ | 0.1087 | 0.09503 | 0.0137 | 0.07213 | 7 | 7 | -0.2233 to 0.2507 | ns |
| Construct 6^1-314^ vs. WT-TDP-GFP^1-414^ | 0.1087 | 0.09588 | 0.01285 | 0.07213 | 7 | 7 | -0.2241 to 0.2498 | ns |
| Construct 6^1-314^ vs. td-tomatoTDP43^1-414^ | 0.1087 | 0.2183 | -0.1096 | 0.06801 | 7 | 9 | -0.3331 to 0.1138 | ns |
| Construct 6^1-314^ vs. Actin GFP | 0.1087 | 0.09233 | 0.0164 | 0.07508 | 7 | 6 | -0.2303 to 0.2631 | ns |
| Construct 6^1-314^ vs. CD63 GFP | 0.1087 | 0.07015 | 0.03858 | 0.07508 | 7 | 6 | -0.2081 to 0.2852 | ns |
| Construct 6^1-314^ vs. pcDNA GFP | 0.1087 | 0.1752 | -0.06648 | 0.07508 | 7 | 6 | -0.3131 to 0.1802 | ns |
| Construct 6^1-314^ vs. WT Unlabeled | 0.1087 | 0.1005 | 0.008216 | 0.06801 | 7 | 9 | -0.2152 to 0.2317 | ns |
| Construct 10^1-105^ vs. WT-TDP-GFP^1-414^ | 0.09503 | 0.09588 | -0.0008499 | 0.07213 | 7 | 7 | -0.2378 to 0.2361 | ns |
| Construct 10^1-105^ vs. td-tomatoTDP43^1-414^ | 0.09503 | 0.2183 | -0.1233 | 0.06801 | 7 | 9 | -0.3467 to 0.1001 | ns |
| Construct 10^1-105^ vs. Actin GFP | 0.09503 | 0.09233 | 0.002698 | 0.07508 | 7 | 6 | -0.2440 to 0.2494 | ns |
| Construct 10^1-105^ vs. CD63 GFP | 0.09503 | 0.07015 | 0.02488 | 0.07508 | 7 | 6 | -0.2218 to 0.2715 | ns |
| Construct 10^1-105^ vs. pcDNA GFP | 0.09503 | 0.1752 | -0.08018 | 0.07508 | 7 | 6 | -0.3268 to 0.1665 | ns |
| Construct 10^1-105^ vs. WT Unlabeled | 0.09503 | 0.1005 | -0.005482 | 0.06801 | 7 | 9 | -0.2289 to 0.2180 | ns |
| WT-TDP-GFP^1-414^ vs. td-tomatoTDP43^1-414^ | 0.09588 | 0.2183 | -0.1225 | 0.06801 | 7 | 9 | -0.3459 to 0.1010 | ns |
| WT-TDP-GFP^1-414^ vs. Actin GFP | 0.09588 | 0.09233 | 0.003548 | 0.07508 | 7 | 6 | -0.2431 to 0.2502 | ns |
| WT-TDP-GFP^1-414^ vs. CD63 GFP | 0.09588 | 0.07015 | 0.02573 | 0.07508 | 7 | 6 | -0.2209 to 0.2724 | ns |
| WT-TDP-GFP^1-414^ vs. pcDNA GFP | 0.09588 | 0.1752 | -0.07933 | 0.07508 | 7 | 6 | -0.3260 to 0.1673 | ns |
| WT-TDP-GFP^1-414^ vs. WT Unlabeled | 0.09588 | 0.1005 | -0.004632 | 0.06801 | 7 | 9 | -0.2281 to 0.2188 | ns |
| td-tomatoTDP43^1-414^ vs. Actin GFP | 0.2183 | 0.09233 | 0.126 | 0.07112 | 9 | 6 | -0.1077 to 0.3597 | ns |
| td-tomatoTDP43^1-414^ vs. CD63 GFP | 0.2183 | 0.07015 | 0.1482 | 0.07112 | 9 | 6 | -0.08548 to 0.3819 | ns |
| td-tomatoTDP43^1-414^ vs. pcDNA GFP | 0.2183 | 0.1752 | 0.04313 | 0.07112 | 9 | 6 | -0.1905 to 0.2768 | ns |
| td-tomatoTDP43^1-414^ vs. WT Unlabeled | 0.2183 | 0.1005 | 0.1178 | 0.06362 | 9 | 9 | -0.09117 to 0.3268 | ns |
| Actin GFP vs. CD63 GFP | 0.09233 | 0.07015 | 0.02218 | 0.07791 | 6 | 6 | -0.2338 to 0.2782 | ns |
| Actin GFP vs. pcDNA GFP | 0.09233 | 0.1752 | -0.08288 | 0.07791 | 6 | 6 | -0.3389 to 0.1731 | ns |
| Actin GFP vs. WT Unlabeled | 0.09233 | 0.1005 | -0.00818 | 0.07112 | 6 | 9 | -0.2419 to 0.2255 | ns |
| CD63 GFP vs. pcDNA GFP | 0.07015 | 0.1752 | -0.1051 | 0.07791 | 6 | 6 | -0.3610 to 0.1509 | ns |
| CD63 GFP vs. WT Unlabeled | 0.07015 | 0.1005 | -0.03036 | 0.07112 | 6 | 9 | -0.2640 to 0.2033 | ns |
| pcDNA GFP vs. WT Unlabeled | 0.1752 | 0.1005 | 0.0747 | 0.07112 | 6 | 9 | -0.1590 to 0.3084 | ns |

Supplementary Table S8. ANOVA table corresponding to Figure 4C. The mean values provided here are the percentage of td-tomatoTDP43^1-414^ acceptor cells that are double labeled (acceptor cells with positive transfer).

| **Test details** | **Mean 1** | **Mean 2** | **Mean Diff.** | **SE of diff.** | **n1** | **n2** | **95% CI of diff.** | **Summary** |
| --- | --- | --- | --- | --- | --- | --- | --- | --- |
| Construct 2^86-414^ vs. Construct 5^257-414^ | 0.02245 | 0.01734 | 0.005117 | 0.01118 | 3 | 3 | -0.03360 to 0.04383 | ns |
| Construct 2^86-414^ vs. Construct 6^1-314^ | 0.02245 | 0.0191 | 0.003348 | 0.01118 | 3 | 3 | -0.03537 to 0.04207 | ns |
| Construct 2^86-414^ vs. Construct 10^1-105^ | 0.02245 | 0.01335 | 0.009098 | 0.01118 | 3 | 3 | -0.02962 to 0.04782 | ns |
| Construct 2^86-414^ vs. WT-TDP-GFP^1-414^ | 0.02245 | 0.01826 | 0.004191 | 0.01118 | 3 | 3 | -0.03453 to 0.04291 | ns |
| Construct 2^86-414^ vs. Actin GFP | 0.02245 | 0.01452 | 0.007928 | 0.01118 | 3 | 3 | -0.03079 to 0.04665 | ns |
| Construct 2^86-414^ vs. CD63 GFP | 0.02245 | 0.03264 | -0.01019 | 0.01118 | 3 | 3 | -0.04890 to 0.02853 | ns |
| Construct 2^86-414^ vs. WT Unlabeled | 0.02245 | 0.02006 | 0.002392 | 0.01118 | 3 | 3 | -0.03633 to 0.04111 | ns |
| Construct 5^257-414^ vs. Construct 6^1-314^ | 0.01734 | 0.0191 | -0.001769 | 0.01118 | 3 | 3 | -0.04049 to 0.03695 | ns |
| Construct 5^257-414^ vs. Construct 10^1-105^ | 0.01734 | 0.01335 | 0.003981 | 0.01118 | 3 | 3 | -0.03474 to 0.04270 | ns |
| Construct 5^257-414^ vs. WT-TDP-GFP^1-414^ | 0.01734 | 0.01826 | -0.0009258 | 0.01118 | 3 | 3 | -0.03964 to 0.03779 | ns |
| Construct 5^257-414^ vs. Actin GFP | 0.01734 | 0.01452 | 0.002811 | 0.01118 | 3 | 3 | -0.03591 to 0.04153 | ns |
| Construct 5^257-414^ vs. CD63 GFP | 0.01734 | 0.03264 | -0.0153 | 0.01118 | 3 | 3 | -0.05402 to 0.02341 | ns |
| Construct 5^257-414^ vs. WT Unlabeled | 0.01734 | 0.02006 | -0.002725 | 0.01118 | 3 | 3 | -0.04144 to 0.03599 | ns |
| Construct 6^1-314^ vs. Construct 10^1-105^ | 0.0191 | 0.01335 | 0.00575 | 0.01118 | 3 | 3 | -0.03297 to 0.04447 | ns |
| Construct 6^1-314^ vs. WT-TDP-GFP^1-414^ | 0.0191 | 0.01826 | 0.000843 | 0.01118 | 3 | 3 | -0.03787 to 0.03956 | ns |
| Construct 6^1-314^ vs. Actin GFP | 0.0191 | 0.01452 | 0.00458 | 0.01118 | 3 | 3 | -0.03414 to 0.04330 | ns |
| Construct 6^1-314^ vs. CD63 GFP | 0.0191 | 0.03264 | -0.01353 | 0.01118 | 3 | 3 | -0.05225 to 0.02518 | ns |
| Construct 6^1-314^ vs. WT Unlabeled | 0.0191 | 0.02006 | -0.0009564 | 0.01118 | 3 | 3 | -0.03967 to 0.03776 | ns |
| Construct 10^1-105^ vs. WT-TDP-GFP^1-414^ | 0.01335 | 0.01826 | -0.004907 | 0.01118 | 3 | 3 | -0.04362 to 0.03381 | ns |
| Construct 10^1-105^ vs. Actin GFP | 0.01335 | 0.01452 | -0.00117 | 0.01118 | 3 | 3 | -0.03989 to 0.03755 | ns |
| Construct 10^1-105^ vs. CD63 GFP | 0.01335 | 0.03264 | -0.01928 | 0.01118 | 3 | 3 | -0.05800 to 0.01943 | ns |
| Construct 10^1-105^ vs. WT Unlabeled | 0.01335 | 0.02006 | -0.006706 | 0.01118 | 3 | 3 | -0.04542 to 0.03201 | ns |
| WT-TDP-GFP^1-414^ vs. Actin GFP | 0.01826 | 0.01452 | 0.003737 | 0.01118 | 3 | 3 | -0.03498 to 0.04245 | ns |
| WT-TDP-GFP^1-414^ vs. CD63 GFP | 0.01826 | 0.03264 | -0.01438 | 0.01118 | 3 | 3 | -0.05309 to 0.02434 | ns |
| WT-TDP-GFP^1-414^ vs. WT Unlabeled | 0.01826 | 0.02006 | -0.001799 | 0.01118 | 3 | 3 | -0.04052 to 0.03692 | ns |
| Actin GFP vs. CD63 GFP | 0.01452 | 0.03264 | -0.01811 | 0.01118 | 3 | 3 | -0.05683 to 0.02060 | ns |
| Actin GFP vs. WT Unlabeled | 0.01452 | 0.02006 | -0.005536 | 0.01118 | 3 | 3 | -0.04425 to 0.03318 | ns |
| CD63 GFP vs. WT Unlabeled | 0.03264 | 0.02006 | 0.01258 | 0.01118 | 3 | 3 | -0.02614 to 0.05130 | ns |

Supplementary Table S9. ANOVA table corresponding to Figure 4D. The mean values provided here are the percentage of GFP-tagged protein expressing acceptor cells that are double labeled (acceptor cells with positive transfer).

| **Test details** | **Mean 1** | **Mean 2** | **Mean Diff.** | **SE of diff.** | **n1** | **n2** | **95% CI of diff.** | **Summary** |
| --- | --- | --- | --- | --- | --- | --- | --- | --- |
| Construct 2^86-414^ vs. Construct 5^257-414^ | 0.08346 | 0.2523 | -0.1688 | 0.06267 | 3 | 3 | -0.3828 to 0.04517 | ns |
| Construct 2^86-414^ vs. Construct 6^1-314^ | 0.08346 | 0.05537 | 0.02809 | 0.06267 | 3 | 3 | -0.1859 to 0.2421 | ns |
| Construct 2^86-414^ vs. Construct 10^1-105^ | 0.08346 | 0.06162 | 0.02184 | 0.06267 | 3 | 3 | -0.1922 to 0.2358 | ns |
| Construct 2^86-414^ vs. WT-TDP-GFP^1-414^ | 0.08346 | 0.06597 | 0.01749 | 0.06267 | 3 | 3 | -0.1965 to 0.2315 | ns |
| Construct 2^86-414^ vs. Actin GFP | 0.08346 | 0.1585 | -0.07501 | 0.06267 | 3 | 3 | -0.2890 to 0.1390 | ns |
| Construct 2^86-414^ vs. CD63 GFP | 0.08346 | 0.1519 | -0.06842 | 0.06267 | 3 | 3 | -0.2824 to 0.1456 | ns |
| Construct 5^257-414^ vs. Construct 6^1-314^ | 0.2523 | 0.05537 | 0.1969 | 0.06267 | 3 | 3 | -0.01708 to 0.4109 | ns |
| Construct 5^257-414^ vs. Construct 10^1-105^ | 0.2523 | 0.06162 | 0.1907 | 0.06267 | 3 | 3 | -0.02333 to 0.4047 | ns |
| Construct 5^257-414^ vs. WT-TDP-GFP^1-414^ | 0.2523 | 0.06597 | 0.1863 | 0.06267 | 3 | 3 | -0.02768 to 0.4003 | ns |
| Construct 5^257-414^ vs. Actin GFP | 0.2523 | 0.1585 | 0.09382 | 0.06267 | 3 | 3 | -0.1202 to 0.3078 | ns |
| Construct 5^257-414^ vs. CD63 GFP | 0.2523 | 0.1519 | 0.1004 | 0.06267 | 3 | 3 | -0.1136 to 0.3144 | ns |
| Construct 6^1-314^ vs. Construct 10^1-105^ | 0.05537 | 0.06162 | -0.006254 | 0.06267 | 3 | 3 | -0.2203 to 0.2077 | ns |
| Construct 6^1-314^ vs. WT-TDP-GFP^1-414^ | 0.05537 | 0.06597 | -0.0106 | 0.06267 | 3 | 3 | -0.2246 to 0.2034 | ns |
| Construct 6^1-314^ vs. Actin GFP | 0.05537 | 0.1585 | -0.1031 | 0.06267 | 3 | 3 | -0.3171 to 0.1109 | ns |
| Construct 6^1-314^ vs. CD63 GFP | 0.05537 | 0.1519 | -0.09651 | 0.06267 | 3 | 3 | -0.3105 to 0.1175 | ns |
| Construct 10^1-105^ vs. WT-TDP-GFP^1-414^ | 0.06162 | 0.06597 | -0.004349 | 0.06267 | 3 | 3 | -0.2184 to 0.2097 | ns |
| Construct 10^1-105^ vs. Actin GFP | 0.06162 | 0.1585 | -0.09685 | 0.06267 | 3 | 3 | -0.3108 to 0.1172 | ns |
| Construct 10^1-105^ vs. CD63 GFP | 0.06162 | 0.1519 | -0.09026 | 0.06267 | 3 | 3 | -0.3043 to 0.1237 | ns |
| WT-TDP-GFP^1-414^ vs. Actin GFP | 0.06597 | 0.1585 | -0.0925 | 0.06267 | 3 | 3 | -0.3065 to 0.1215 | ns |
| WT-TDP-GFP^1-414^ vs. CD63 GFP | 0.06597 | 0.1519 | -0.08591 | 0.06267 | 3 | 3 | -0.2999 to 0.1281 | ns |
| Actin GFP vs. CD63 GFP | 0.1585 | 0.1519 | 0.006591 | 0.06267 | 3 | 3 | -0.2074 to 0.2206 | ns |
